# Gender, climate and landowning: Sources of variability in the weather pattern change and ideal fertility relationship in Sahelian West Africa

**DOI:** 10.1553/p-pfaj-9kzm

**Published:** 2024-04-30

**Authors:** Isabel H. McLoughlin Brooks

**Affiliations:** 1 Population Research Center & Department of Sociology, University of Texas at Austin, Austin, TX, USA

**Keywords:** Temperature, Precipitation, Sahel, West Africa, Gender composition, Ideal family size

## Abstract

This paper advances our understanding of the relationship between climate change and ideal fertility in Sahelian West Africa by exploring sources of variation in that relationship. Using an integrated dataset of Demographic and Health Surveys with monthly rainfall and temperature data, the analyses model dimensions of prospective ideal fertility for young, childless men and women in Senegal, Mali, Burkina Faso and Nigeria. Temperature, particularly in the arid climate zone, is shown to have a positive effect on ideal fertility. Landowning insulates individuals from adjusting their fertility ideals in response to change. Gender-stratified models reveal that under hotter conditions, women have a higher ideal number of children but their ideal gender composition remains relatively balanced, while men do not change their ideal number of children but show a preference for more sons. The increase in ideal fertility in response to weather change may be understood as an increasing need to generate human capital to meet the increased labour demands that climate change brings over both the short and the long term.

## Introduction

A fertility response – either having or limiting births – is a well-established way that individuals or households react to resource constraints (Eissler et al., 2019; Grace, 2017; Sobotka et al., 2011). However, since actual fertility levels are a product of a number of stochastic processes (Bongaarts and Potter, 2013), achieving one’s expected fertility is not guaranteed. Therefore, the prospective ideal number of children, which represents an underlying demand for children, is a more apt indicator of people’s reactions to changes in their circumstances. Climate change is a serious threat to people’s livelihoods and can substantially affect their circumstances. Populations in West Africa are particularly vulnerable to the hotter temperatures and erratic rainfall conditions arising from climate change (Lee et al., 2023; Niang et al., 2014; Schmidhuber and Tubiello, 2007), largely because most people in the region rely on rain-fed subsistence agriculture (Goedde et al., 2019), and everyone exists within food and economic systems that rely on the successful production of local agricultural goods and livestock (Grace, 2017).

In the rural livelihoods framework (Curran, 2022; de Sherbinin et al., 2008; Ellis, 1998, 2000), households, as the units of production and reproduction, pursue many different activities to survive by mobilising various types of capital (natural, social, physical, financial and human) (de Sherbinin et al. 2008). One way that the ideal number of children can be understood within this framework is as a particular type of investment in human capital. Weather pattern change most directly affects natural capital, or local environmental conditions. When people experience a strain on natural capital, they may act to balance their household resources by investing in other forms of capital, including human capital (de Sherbinin et al., 2008). In forming their fertility ideals, young adults may reflect on their household’s labour needs, as well as on the socio-cultural norms about family and childrearing, the resources at their disposal and the changes in conditions they have experienced. Considering this, I ask: How many children do young, childless adults consider to be ideal in the face of changing weather patterns? I look for variation in this relationship across several dimensions by asking the following questions: Are fertility ideals affected by weather change differently in different climates? How do these effects vary by the household’s physical capital (e.g., ownership of agricultural land or livestock) or the adult’s gender? Does the gender composition of young adults’ ideal family vary with weather change? Finally, do temperature and precipitation change have different effects? To test these questions, I integrate detailed weather data at the community level with georeferenced Demographic and Health Survey (DHS) data collected over 10 years in Senegal, Mali, Burkina Faso and Nigeria.

This work advances existing scholarship in several ways. First, rather than exploring the consequences of climate change for fertility or fertility-related behaviours, it emphasises its consequences for underlying fertility ideals. In so doing, it offers new insights into the upstream, psychosocial responses to increasing climate uncertainty in Sahelian West Africa. Second, whereas past work has explored variation in the effects of climate change across administrative regions that do not necessarily map onto climatic conditions in a meaningful way, this study analyses variation across climate zones. Thus, it more accurately represents the physical environment from which people derive their natural resources. Third, to better understand potential variation across different types of physical capital, this study explores heterogeneity in the weather change–fertility relationship by land or livestock ownership. Ownership of these assets may indicate how vulnerable to climate change households are given their reliance on these assets and may provide a more accurate assessment than analyses that rely on remotely sensed or estimated geographic livelihood zones. Lastly, this work explores variation in these effects by gender. Men are largely absent from studies of fertility in general, and especially in African settings (Dodoo and Frost, 2008; for recent exceptions see Smith, 2020; Grant and Curtis, 2022). Given the gendered roles in agriculture and in household and fertility decision-making (Andriano et al., 2021; Ezeh, 1993; Bankole, 1995), understanding men’s, in addition to women’s, ideals related to family size and the sex of children is vital to understanding how fertility change will progress in the years to come. This may be one of the first studies that investigates fertility ideals (in total and by gender composition) for women and men in relation to changing weather patterns.

## Background

### Rural livelihoods framework and ideal family size

The rural livelihoods framework describes how households in rural or agricultural economies must rely on a diverse set of activities to survive, such as farming, herding or off-farm employment (de Sherbinin et al., 2008; Ellis, 1998, 2000). Household members mobilise their available assets to engage in these activities. These assets can take many forms, including natural capital (local environment, e.g., water, forest, soil), social capital (e.g., networks, groups), human capital (e.g., education, local knowledge, people, workers), physical capital (e.g., land, livestock) and financial capital (e.g., cash, available credit). Households typically have a combination of these assets at their disposal, which may be ever-shifting. Investment in one area may require the depletion or rebalancing of others. In West Africa and other rural areas, the main forms of capital households rely on are weather-dependent (Goedde et al., 2019). In such a system, weather pattern change can disrupt natural capital, requiring households to re-balance or prioritise other types of capital to support themselves and to plan for the future. These efforts to stabilise household resources can take different forms, some of which may involve generating human capital (e.g., fertility responses), and may also be informed by cultural expectations about children, family and gender roles.

Across West Africa, child labour serves as a means for increased household production and to instruct children in the livelihood skills they need to support themselves in the future (Busquet et al., 2021). Thus, performing labour is considered a vital part of a child’s social integration into society (Adonteng-Kissi, 2018; Busquet et al., 2021; Twum-Danso, 2009). While childrearing is costly in the short term, especially as the costs of supporting children rise, children participate in household livelihood tasks from an early age. Parents gradually scale up their children’s livelihood tasks based on age and ability (Busquet et al., 2021). By age five, children can usually assist adults in tending crops (Bass, 2004). By age 12 for girls and by age 14 for boys, children are considered equal to adults in terms of their ability to work (ibid). Over the long term, children are expected to provide financial security and to care for their parents in old age (Stecklov, 1997).

Beyond having productive value, children bring social and affective benefits to their parents. Marriage and childbearing are nearly universal in sub-Saharan Africa, and especially in the Sahel (Shapiro and Gebreselassie, 2014). Kinship relations are strong in West Africa; marriage, or the bringing together of kin groups, and childbearing, or the continuation of one’s lineage, are important milestones (Bawah et al., 1999; Caldwell and Caldwell, 1987; Smith, 2020). Children provide socio-cultural benefits to their parents by ensuring the “competent masculinity” of the father (Smith, 2020, p. 107) or by solidifying the social position and relationships of the mother (Bledsoe and Pison, 1994; Hollos et al., 2009). Young people in West Africa typically feel social pressure to have at least a few children; a “few” may be at least four children in some parts of the region (Smith, 2004, 2020).

A person’s prospective fertility ideal – i.e., their ideal number of children *before* they have begun childbearing – is likely shaped in part by how they view their future, and generally reflects the prevailing social norms about family and children. Voluntary childlessness in this region is rare, at less than 0.02 per cent (Verkroost and Monden, 2022). Thus, given the productive, affective and social benefits of having children in this region, young people in West Africa are likely to consider having at least a few children ideal. The exact number tends to reflect the number of children they expect to need to ensure household survival and to support them in their old age, as well as how many children they can support. In the context of climate change, ideal fertility also tends to be reflective of anticipated coping strategies (Brauner-Otto, 2014). Changes in household resources may change perceptions about children and their associated costs and benefits (Thiede, 2022). Children often contribute to the household by completing tasks that may become more time-consuming after climate change impacts the land. For this reason, children’s labour may be particularly valuable during periods of natural capital resource strain (Beegle et al., 2006). Children’s labour frees adults, and particularly women, from doing such work (Cain, 1977). Thus, changing weather patterns may influence how young people envision their family size, suggesting that they will need a larger number of children than they would under average conditions.

However, it is also plausible that environmental and household resource stress will reduce the demand for children and will therefore encourage a smaller ideal family size. If the household cannot support as many children, or if the degradation of environmental conditions makes resources scarce, then, ceteris paribus, the value of children’s labour in collecting those scarce resources would decline (Biddlecom et al., 2005). Recent studies conducted in Nigeria and Malawi have suggested that economic uncertainty or environmental shocks can encourage young adults to delay parenthood (Smith, 2020) or postpone further childbearing (Grant and Curtis, 2022). When they have fewer or lower quality natural capital resources at their disposal, young adults might anticipate having fewer resources to rear children in the short and the long term and may therefore see having a smaller family as more advantageous. Eissler et al. (2019) found that in West Africa, the ideal family size was predicted to be lower when people were experiencing negative precipitation anomalies.

A number of scholars have explored the concept of ideal family size; its usefulness in predicting behaviour, such as contraceptive use or subsequent birth rates (Coombs, 1974; Westoff et al., 1957); and, more recently, its dynamism (Hayford, 2009; Trinitapoli and Yeatman, 2018; Yeatman et al., 2013; Yeatman and Sennott, 2014). The literature uses various terms to describe family size preferences, such as expected, desired or ideal family size. These concepts may have slightly different semantic meanings, but are all “tapping a common psychological orientation” (Ní Bhrolcháin and Beaujouan, 2019, p. 31) related to the future number of children. Fertility preferences tend to vary over the life course (Hayford, 2009), reflecting both personal and broader, community-level circumstances or experiences (Schoen et al., 1999; Sennott and Yeatman, 2012; Trinitapoli and Yeatman, 2011, 2018; Yeatman et al., 2013). People’s preferences are particularly flexible at younger ages (Iacovou and Tavares, 2011; Yeatman et al., 2013), and tend to change as family formation becomes within reach (Schoen et al., 1999; Yeatman et al., 2013). Nonetheless, people’s fertility preferences may be predictive of their behaviour in the short term (Hayford, 2009). Moreover, in this region, family size ideals are strongly correlated with total fertility rates at the population level (Bongaarts and Casterline, 2013). The prospective ideal number of children of young adults is likely to capture preferences that drive their behaviour in the near future, which coincide with major life course transitions such as marriage (DHS StatCompiler, 2023a) and, quickly thereafter, first childbearing (DHS StatCompiler, 2023b). While young adults may lack exact information about or experience with the actual costs and benefits of childrearing, there is evidence that the *perceived* costs of having children have a large impact on their desired fertility (Mason, 1997).

### Environmental conditions and fertility impacts

The relationship between the local environment and fertility is not universal. Depending on the context, poor environmental conditions or a negative change in access to natural resources may, for example, yield an increase in the preferred family size (Biddlecom et al., 2005; Brauner-Otto and Axinn, 2017; Eissler et al., 2019), the desire to have another child in the future (Sasson and Weinreb, 2017) or a lower likelihood of using contraception (Brauner-Otto, 2014). In the rural livelihood framework, households that invest in generating human capital by having (many) children typically do not have other types of capital at their disposal to insulate them from poor environmental conditions or some kind of negative change. In such contexts, it is often the poorest households that focus their efforts on childbearing (Sasson and Weinreb, 2017). When the land is degraded and resources are constrained, additional labour may be needed to generate sufficient crops, firewood, water or fodder. Given that land degradation may increase the demand for children who can provide labour, the demand for children should rise when environmental strain increases (Bhandari and Ghimire, 2013; Biddlecom et al., 2005; Brauner-Otto, 2014; Brauner-Otto and Axinn, 2017; Dasgupta, 1995; de Sherbinin et al., 2008; Filmer and Pritchett, 2002; Lutz and Scherbov, 2000). Childbearing, at least in these contexts, is seen as advantageous, as children’s labour can help households exploit the remaining natural capital resources.

In other contexts, a negative change in environmental conditions can encourage lower actual fertility (e.g., pregnancies or births) (Alam and Pörtner, 2018), reduce the desire to have a child (Brooks et al., 2023; Eissler et al., 2019) or reduce the ideal family size (Eissler et al., 2019). In Kenya, a poor growing season lowered the desire for children in the short term (Brooks et al., 2023); and in Tanzania, crop losses are associated with higher contraception use (Alam and Pörtner, 2018). In some contexts, this negative affect is concentrated in poorer or less endowed households. In Indonesia, for instance, temperature shocks have been linked to reduced fertility intentions among farming households and less educated women (Sellers and Gray, 2019). Thus, fertility responses are varied and context-dependent.

### Weather context by climate

**Figure 1:**
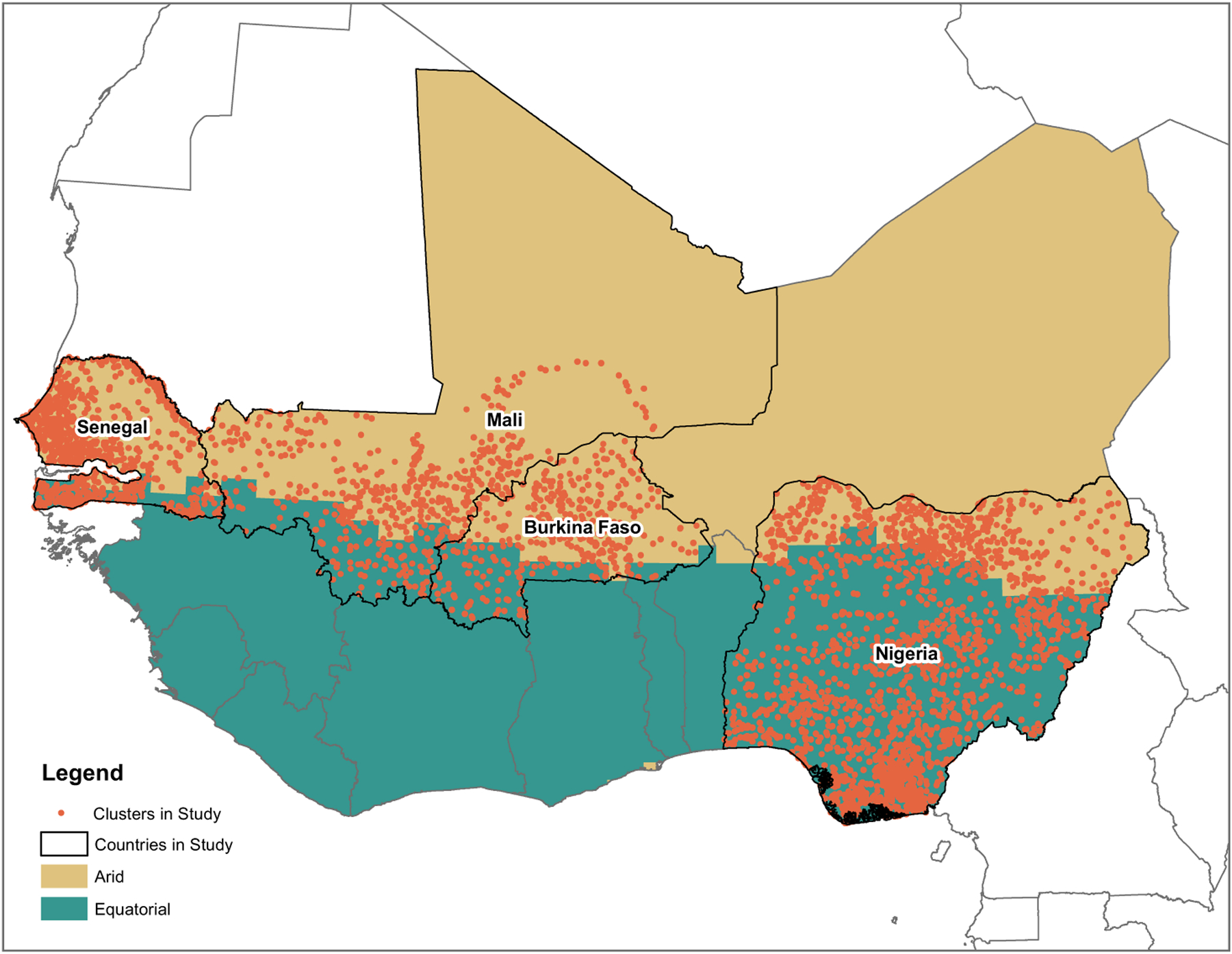
Map of climate zones and Demographic and Health Survey cluster locations in the analytic sample

The effects of environmental conditions vary across regions (Eissler et al., 2019) and country boundaries (Brooks et al., 2023) within sub-Saharan Africa. There are other geographical sources of variation that may also be salient. West Africa is a climatically diverse region. Thus, considering the region as a whole obscures potential variation in the natural and physical capital resources available. Moving from north to south, the climate shifts from arid to equatorial (Köppen and Geiger, 2020). See Figure 1 for a map of the countries included in this study and the delineations of each climate zone within the countries’ boundaries.

The arid climate zone is characterised by hot, dry weather and desert and steppe landscapes. Temperatures are high all year round in these areas, with average peaks reaching 40 degrees Celsius (Tappan et al., 2016; Steel et al., 2023). The desert receives only minimal rainfall, under 200mm annually, which falls in a concentrated period lasting under two months. The steppe gets between 500 and 600mm of rain annually over a five-month period (USAID, 2018). By the end of the 21st century, an increase in the average temperature of up to six degrees Celsius is expected in this zone (Niang et al., 2014), which will lead to more erratic rainfall patterns (Lee et al., 2023), including both negative and positive changes in rainfall amounts (Niang et al., 2014). In addition to recurrent drought and expanding desertification, the arid climate zone suffers from poor soil and short growing seasons (Kpadonou et al., 2017). The staple crops here are produced for subsistence with little input and are less vulnerable to small weather deviations (OECD/SWAC, 2009).

The equatorial climate zone is nearest to the equator and includes tropical rainforests and savanna landscapes. Within this climate zone there are three sub-climate zones with distinct seasonal rainfall patterns. The varying wet seasons receive from 700 mm to over 1800 mm of rain annually (Steel et al., 2023; Tappan et al., 2016). Average temperatures are warm in the equatorial climate zone, never dropping below 18 degrees Celsius (Tappan et al., 2016). In this zone, increases in temperature are likely to be between two and four degrees Celsius by the end of the 21st century (Niang et al., 2014), which will increase the incidence of floods and droughts (Lee et al., 2023). The sustained periods of rain and the fertile soil in this climate zone allow for significant and diverse crop cultivation. However, moving south within the zone, the crops that are grown become increasingly water-intensive (OECD/SWAC, 2009) and irrigation is extremely limited (World Bank, 2020). In addition to staple food crops, cash and export crops are grown in this zone (OECD/SWAC, 2014).

These climate-specific conditions suggest that individuals living in different climate zones may interpret weather conditions and changes differently and may thus have different ideals related to family size. This association is not commonly considered in environmental and fertility research. In one notable exception, fertility timing was found to vary in response to rainfall patterns only in the dry climates of rural Mexico, and not in the humid ones (Simon, 2017).

### Physical capital, agricultural practices and fertility

Household resources, as they relate to the way individuals or households make their living, may also modify the relationship between weather change and fertility. Ownership of livestock or of agricultural land implies some degree of reliance on these assets for livelihood pursuits (animal husbandry or crop cultivation), and may therefore influence notions of family size based on the household labour needs associated with these different livelihoods.

There are clear empirical differences by livelihood in fertility outcomes, such as in the total fertility rate (Grace and Nagle, 2015) or the desire or intention to have a child (Sellers and Gray, 2019). For example, in Indonesia, delays in the onset of the monsoon season, which can affect rice planting, were found to be associated with an increase in the intention to have another child among non-agricultural households, but with a decrease in this intention among farming households (Sellers and Gray, 2019). This notable contrast amid similar environmental conditions is a reason to suspect that the households’ agricultural pursuits and/or the physical capital resources at their disposal are important factors in their fertility intentions. In an example from Mali based on livelihood zones,^1^ the total fertility rate and the desire for another child was found to decline across the crop cultivation, agropastoralist (crop cultivation and animal herding) and pastoralist (animal herding) livelihood zones (Grace and Nagle, 2015). Although not everyone living in a given zone is engaged in that particular practice, this overall pattern suggests that livelihood type matters for fertility and its psychosocial antecedents.

Animals provide eggs, meat or dairy products, or they are beasts of burden. Animal husbandry can take several forms, which vary by season, animal type, herd size or the amount of time, labour or money available to be invested in their care; with the most intensive forms (pastoralism and transhumance) involving the seasonal movement of herds and people (Turner and Hiernaux, 2008). Young men are preferred for herding labour, which is often hired out (ibid). The fertility of pastoralists has historically been lower than that of settled cultivators (Henin, 1968, 1969; Meir, 1987; Borgerhoff Mulder, 1992; Swift, 1977). This is likely because livestock ownership requires less labour and small populations are therefore advantageous to pastoralists’ survival (Swift, 1977). As a type of physical capital, livestock are movable and easily sold, unlike land, which is fixed (Loker, 1993; Moll, 2005; Borgerhoff Mulder, 1992). While the movability of livestock may make pastoralists somewhat more resilient to local climate change than cultivators who are fixed in place, they are vulnerable to the weather changes, desertification and land use changes that restrict available pastureland over a broader area (Bekele et al., 2022).

Subsistence farming often produces low yields, with most households growing staple crops that require basic inputs on small amounts of land. Thus, food insecurity is high among subsistence farmers (Giller et al., 2021). As crop cultivation is labour-intensive and responds to increased inputs (e.g., more workers or fertiliser), cultivators tend to have higher fertility (Grace and Nagle, 2015). However, the land security hypothesis (Stokes and Schutjer, 1984) argues that actually owning the land that they farm creates economic security for cultivators, which, in turn, lowers their demand for children (Carr et al., 2006; Stokes and Schutjer, 1984), because their need for financial support in old age is somewhat reduced (Bhandari and Ghimire, 2013).

### Gender differences in fertility preferences, environmental interaction and agricultural practices

For young men and women living in sub-Saharan Africa today, conceptualising one’s future family size is somewhat fraught because of the opposing forces of “traditional” cultural pronatalism and the “modern” realities of increasingly expensive investments in children, which may feel out of reach in uncertain times (Agadjanian, 2005; Smith, 2004, 2020). Children are highly valued and bring social benefits to their parents (Agadjanian, 2005; Bledsoe, 1980; Bledsoe and Pison, 1994; Hollos et al., 2009; Smith, 2004, 2020), but the parental responsibilities of men and women differ, and may produce distinct ideals related to family size. Men feel more financial responsibility for children (Manuh, 1997; Smith, 2020), and this pressure may encourage the postponement of childbearing or a smaller desired family size among men (Smith, 2004, 2020). However, the desire to meet social expectations and to generate “wealth in people” may keep the ideal family size high for men. Women are the primary caregivers for young children, which places the burden and the reality of birthing and caring for large families on them (and their bodies) (Agadjanian, 2005). Nonetheless, childbearing confers social benefits and status on women, solidifying their marital relationship and their status with their in-laws (Bledsoe and Pison, 1994; Hollos et al., 2009).

These push and pull factors can also stem from environmental circumstances. Disruptions to natural capital, and thus to household resources, will affect men and women in different ways. Men/boys and women/girls interact with the environment in gendered ways (Nightingale, 2006), given their different social positions (Marter-Kenyon et al., 2022; Parpart et al., 2000; van Dijk and Bose, 2016). Moreover, these differences in environmental interaction patterns will vary depending on how environmental stressors influence individuals’ notions of the future, such as their prospective ideal family size and the composition of that ideal family.

Men and women have different levels of access to natural and other resources related to the gendered division of household and agricultural labour (Alesina et al., 2013; Meinzen-Dick et al., 2014) and to gendered differences in power and access to information and opportunity (Marter-Kenyon et al., 2022; Jin et al., 2015; Lambrou and Piana, 2006; Masika, 2002; Silvestri et al., 2012; Terry, 2009). Generally, land tenure rights, inheritance systems and financial capital availability tend to favour men (Fletschner and Kenney, 2014; Quisumbing et al., 2001), giving them more access to and decision-making power over land and physical capital. Thus, men are more likely than women to determine what is grown and what technologies or practices are used (Meijer et al., 2015). In some contexts in which both men and women engage in crop cultivation, they grow different crops. “Men’s crops” are generally more commercially valued, while “women’s crops” are typically used for subsistence, and are more vulnerable to environmental changes (Carr, 2008; Carr and Thompson, 2014; Orr, Homann Kee-Tui, et al., 2016; Orr, Tsusaka, et al., 2016). When working on the same fields, women tend to do more menial tasks like weeding (Orr, Homann Kee-Tui, et al., 2016; Quisumbing et al., 2015) or serve as subordinate labourers (Quisumbing et al., 2015). Women’s interactions with the environment are often related to household subsistence, such as gathering resources to care for the household and the children (Guèye, 2000; Marter-Kenyon et al., 2022). Men and women also occupy and rely on different spaces. For many of their household gathering tasks, women use commonly held land (Agarwal, 1994), which is likely to degrade faster since the users lack individual control over the land and the number of users is often high. Because these tasks are considered “supplemental”, the degradation of these resources is less of a concern (even to women) because it affects women’s time use and not men’s income (Quinn et al., 2003). However, since women’s tasks are needed for household survival, women may still be burdened by environmental change (Bohra-Mishra and Massey, 2011).

Men and women also differ in their environmental risk perceptions and priorities. Due to gendered patterns of engagement with environmental resources, men and women tend to observe different changes and prioritise different actions to mitigate adverse changes (Carr, 2008). In low-income agricultural populations in West Africa, men are more likely than women to perceive risks associated with environmental change (Cullen et al., 2018; Kristjanson et al., 2015) and to express concern about climate change (Badmos et al., 2018), likely due to the gendered division of labour and of income-generating tasks (Quinn et al., 2003) and to differential access to information about the environment (Badmos et al., 2018). Women, by contrast, are more aware than men of scarcity regarding household livelihoods and are more concerned about small-scale adaptation (Briggs et al., 2003).

These differences in environmental interactions may influence the formation of family ideals, including the ideal gender composition of families. Sub-Saharan Africans are not widely known to show sex preference for children. The health or mortality consequences of sex preference in its extreme form that are observed in some Asian contexts are not evident in sub-Saharan Africa (Basu and De Jong, 2010; Jayachandran and Pande, 2017; Kashyap and Behrman, 2020). However, several studies have found evidence of son preference in sub-Saharan African countries (Bongaarts, 2013), specifically among men (Campbell, 1991; Campbell and Campbell, 1997; Mwageni et al., 2001). But there is also evidence that while son preference exists, a relative balance in offspring is considered desirable in these countries (Mwageni et al., 2001). Drivers of son preference may stem from landholding and inheritance systems that favour males (Mwageni et al., 2001), as well as a general dominance of males in decision-making (Andriano et al., 2021). Male children and their potential labour contributions could be seen as more valuable. The wage-earning ability of sons may also mean greater old-age financial security for parents over the long term, further generating a preference for sons.

## Conceptual framework

The connection to natural capital in a rural, agricultural setting, like that of Sahelian West Africa, is strong. Weather pattern change in this setting – particularly hotter temperatures and reduced rainfall – should affect the quality of the natural capital, the household resources and, in turn, the prospective plans of young adults, like their ideal family size. However, whether weather pattern change increases or decreases ideal fertility is context-dependent. Weather patterns that negatively impact agricultural production may result in increased demand for children if the potential returns to children and their labour outweigh the costs, yielding a higher ideal number of children. By contrast, if individuals and their households mobilise other forms of capital to deal with possible strain, or if the costs of children outweigh their potential benefits, then adverse weather changes could either lower the demand for children, which would reduce the ideal number of children, or have no effect, which would leave the ideal number the same as under average conditions. Prospective parents already worry about the availability of financial capital (Smith, 2020); thus, when environmental change engenders more hardship, their perceived ability to support a family may be reduced (Grant and Curtis, 2022).

I explore two types of weather change – temperature and precipitation – to test their potentially differing effects on dimensions of men’s and women’s ideal fertility levels. The existing literature has pointed to the particular effects of (higher) temperature on demographic outcomes (Bakhtsiyarava et al., 2018; Barreca et al., 2018; Bohra-Mishra et al., 2014; Eissler et al., 2019; Gray and Wise, 2016; Riosmena et al., 2018; Thiede et al., 2016). I therefore expect to find that temperature change is generally more influential.

I explore three potential sources of variation in the relationship between the environment and ideal fertility. The first potential source of variation is climate type. In each climate zone, there are different prevailing weather patterns, agricultural conditions and physical conditions. Due to the lack of research on this potential relationship, it remains unclear whether there is an effect, and, if so, what the direction of that effect is within each climate zone. I expect to observe a more pronounced effect of weather change for young adults in the arid climate zone due to its combination of high temperatures, condensed and minimal rainfall and short growing season. This expectation is also related to the larger expected effect of temperature, as temperature deviations in the zone that is already the hottest are likely to be especially impactful. However, it may be reasonable to assume that given the longer growing season(s) in the equatorial climate zone, the livelihoods of the people living there may be more reliant on agriculture production, and changes in established weather patterns could therefore impact their perceptions of the future.

Second, I test the effects of ownership of two types of physical capital. Household ownership of physical capital assets – agricultural land and livestock – implies (to some degree) that the household members rely on these assets for their livelihoods. It is likely that crops or animal products support household subsistence. Ownership of these assets may influence the prospective fertility ideals of young adults in distinct ways because these assets are differentially affected by changes in weather and the agricultural practices they require demand different labour inputs, especially in times of environmental strain. While this paper focuses on young people just starting out in adult life, the ownership of these assets by their natal household could provide a model for balancing agrarian needs and family size, as it might determine whether they inherit land or animals, which could, in turn, influence their expectations about their family size needs.

Land, as a fixed asset, is particularly vulnerable to environmental change in its vicinity – unlike animals, which can move to greener pastures. Crop cultivation requires more labour than animal husbandry. When the environmental conditions are degraded, cultivation requires even more labour, which children typically carry out. Thus, I expect to find that young adults in landowning households have higher ideal fertility than those in landless households. However, the increased labour needed in such households may be somewhat ameliorated by *owning* land, because ownership provides a level of stability, which can substitute for having additional children to provide financial security (Stokes and Schutjer, 1984). Animal husbandry may already be adapted to adverse weather conditions because of seasonal grazing and the ability to move animals to other areas. Livestock do not respond to increased input like farming does. Due to these factors, the effects of adverse weather patterns on livestock owners are not likely to result in an increase in ideal fertility because outputs from livestock do not respond to increased inputs and young children are not a primary source of labour for this livelihood practice.

Lastly, I explore variation in these patterns by gender in two ways: by taking into account the gender of the respondent and the gender composition of the ideal family size. Even within the same household, men and women should have different expectations about the future – including different family ideals – given their different expected roles and privileges. In general, I expect to observe that men have a larger family ideal than women. The social benefits conferred on men when they generate people are large while the immediate burden of caring for children rests on women’s shoulders.

Weather change and the resulting environmental degradation may increase the demand for labour, especially among farming households. Clearing fields, weeding and gathering natural resources are tasks typically performed by women, who could outsource some of the additional labour to children. Therefore, in adverse weather conditions, women may see having a larger number of children as ideal. At the same time, if more agricultural labour is needed due to adverse weather conditions, women may not want to have many children to care for on top of those tasks. In addition to addressing these concerns about labour, previous research has suggested that because of the differences in the spaces they occupy and the information they can access, men and women observe different environmental changes (Carr, 2008), have different levels of concern about those changes (Badmos et al., 2018) and have different plans to deal with those changes (Briggs et al., 2003). It has, for example, been shown that men have more concerns about climate change (Cullen et al., 2018; Kristjanson et al., 2015). These worries may exacerbate men’s existing concerns about their household resources and ability to care for a family, leading them to have a lower ideal number of children. Other research has found that women have more immediate concerns about the short-term future, which may influence their ideals about a future family (Briggs et al., 2003), given that their childbearing is likely to commence soon. In light of these varied pieces of evidence, I treat whether the effect of weather pattern change on fertility ideals varies by gender as an open question.

An important element of idealising a future family may be imagining the gender composition of children. Sex preference is based on gendered social hierarchies or lineage and inheritance systems, to name a few factors, that typically value male over female children. Given the privileges of men in general, especially with regard to controlling environmental and agricultural resources, it is likely that the ideal number of boys will be higher among men, and that changing weather conditions will increase this preference. I expect to find that both men and women show this preference, because boys are more valued in farming work, and both men and women can appreciate the potential benefits of having boys. Despite the preference for assigning boys/men to livestock care tasks, I do not expect to observe a particular effect of weather pattern change for respondents in livestock-owning households, since the practice of animal husbandry does not benefit from increased input and may already be adapted to weather change.

## Data and methods

### Data

To meet the research goals of this paper, I integrated two datasets: the women’s and the men’s Demographic and Health Surveys from Burkina Faso, Mali, Nigeria and Senegal, which were fielded from 2008 through 2018;^2^ and monthly precipitation and temperature data from Climatic Research Unit Time Series data version 4.05.

The Demographic and Health Surveys (DHS) are nationally representative household surveys completed using multistage cluster sampling. The clusters represent a village in rural areas. The DHS provide individual-level and household-level data on women aged 15–49 and men aged 15–64. The surveys are conducted about every four to five years in each country. The surveys included in this sample have geocoded data representing the cluster location. The DHS data were accessed through the Integrated Public Use Microdata Series (IPUMS) DHS dataset, which provides harmonised DHS data across time and geography (Boyle et al., 2022). I have created 10-kilometre “buffers” around each cluster location to determine the weather conditions prevailing around each cluster.^3^

The weather data come from Climatic Research Unit Time Series data version 4.05 (CRUTS4.05), produced by the Climatic Research Unit (CRU) at the University of East Anglia (University of East Anglia Climatic Research Unit et al., 2021). These data are remotely sensed, gridded rasters with a 0.5-degree spatial resolution (about 55 km^2^ grid in West Africa). I use a monthly measure of near-surface temperature in degrees Celsius and monthly precipitation in centimetres for each 10-km cluster buffer to create baseline measures and measures of change.

#### Sample and measures

The sample includes men and women who have not begun childbearing and have not had prior pregnancies^4^ and who are within one standard deviation of the average age at marriage. Thus, all men in the sample are between the ages of 19 and 31, while all women in the sample are between the ages of 15 and 23. I restrict the sample to rural and usual residents, removing visitors.^5^ The final sample consists of 33,732 people living in 3,724 clusters.

The following question is posed to DHS respondents without children: “If you could choose exactly the number of children to have in your whole life, how many would that be?” Asking the question in this way and to this group of young adults removes rationalisation bias related to asking people who already have children to identify their ideal number of children, as they may be unwilling to respond with a lower number than their current parity (Bongaarts, 1990). I include only the numeric responses^6^ to the ideal fertility question. I top-code values of *ideal number of children* (11) through (80) as (10), since that was the largest value with a substantial number of observations. In the subsamples of men and women, I model the ideal number of children by gender of the child. In the DHS, as a follow-up to the main question on the ideal number of children, respondents are asked: “How many of these children would you like to be boys, how many would you like to be girls … ?”^7^ *Ideal number of girls* and *ideal number of boys* each include only the numeric values and are top-coded at 10.

**Figure 2:**
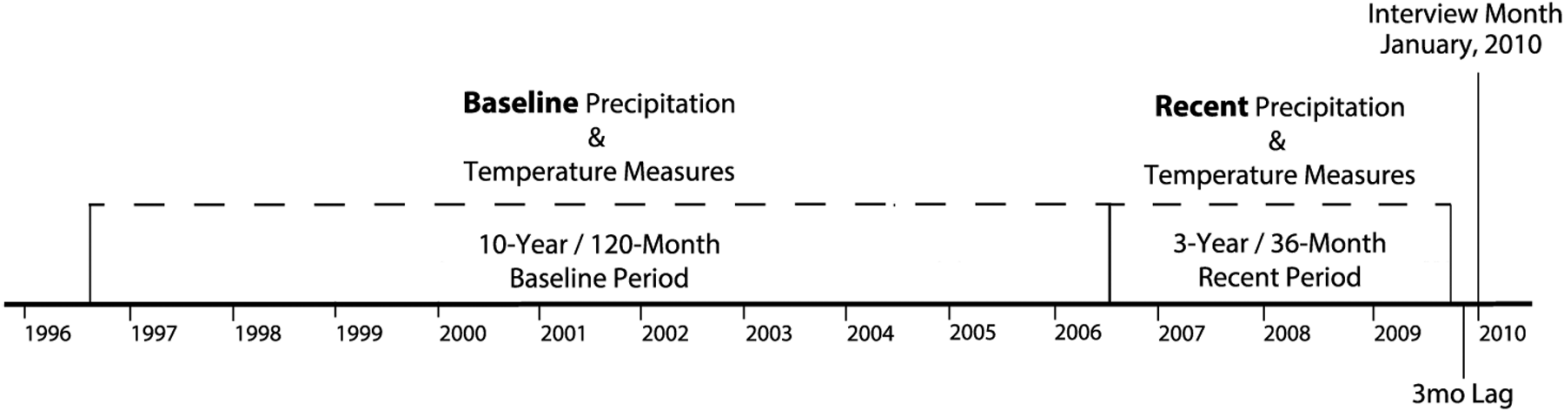
Timeline of weather pattern measures and Demographic and Health Survey interviews

The key explanatory variables are change in precipitation and change in temperature, operationalised as *z-scores* representing change between a 10-year (120-month) baseline mean (that begins 159 months before the DHS interview) and a three-year (36-month) recent mean (with a three-month lag from the interview month). See Figure 2 for a timeline of weather measures and interview timing. The weather change z-scores are measured at the cluster level (cm for precipitation and degrees Celsius for temperature) and are temporally matched to the month of interview for each respondent. The z-scores provide a standard way to describe and understand the positive and negative changes in temperature and precipitation that occur around each cluster. Negative scores mean that rainfall or temperatures were lower in the recent three-year period than in the 10-year baseline, while positive scores mean that rainfall or temperatures were higher in the recent period compared to in the baseline. I include four weather pattern controls: temperature mean and standard deviation and precipitation mean and standard deviation to represent the baseline weather patterns.^8^ In all models I include quadratic versions of the weather change z-scores to account for nonlinearities of the weather pattern change effects.

I delineate the climate zones using the Köppen-Geiger Climate classification system from the World Bank’s data catalogue (Köppen and Geiger, 2020). This system divides the globe into distinct zones based on the main climate description and then by the seasonality and the patterns of precipitation and temperature in each climate zone. West Africa is a vast region that has two distinct main climates: arid and equatorial (see Figure 1). I focus on Burkina Faso, Mali, Nigeria and Senegal because in addition to having available DHS survey data, each of these countries has the two main climate types within their boundaries. The climate represents the natural capital resources available to households and the agricultural capacity of the surrounding area and it dictates the typical range of weather patterns and changes. In the analyses presented here, climate zone is used as a control variable and as a variable by which the models are stratified in a second set of models.

I account for *gender* variation in two ways: first, by variation in the gender of the respondent; and, second, by predicting the ideal number of children by the child’s gender (discussed above). I account for *gender* as a control variable in most models, which is coded as (0) for a male respondent and as (1) for a female respondent. I also use this variable to stratify models to explore the effect of weather change on men’s and women’s ideal fertility outcomes in fully interacted models.

I account for the physical capital resources of household livestock and landownership with two categorical variables. *Household owns livestock* is (1) when the respondent’s household owns any livestock and is (0) otherwise. *Household owns agricultural land* is (1) when the respondent’s household owns agricultural land and is (0) otherwise. In addition to accounting for household physical capital assets, these measures represent some degree of attachment to agricultural livelihood pursuits, such as animal husbandry/pastoralism or crop cultivation, respectively.^9^ These variables are interacted with each of the weather change measures to see how physical asset ownership, or the lack thereof, may affect the relationship between weather change and ideal fertility in the stratified subsample analyses.

The models control for standard demographic variables: age and age squared; a categorical measure of primary level education,^10^ which is (0) if the respondent has 0–5 years of education (incomplete primary) or is (1) if the respondent has 6+ years of education (primary education or more); a binary measure of formal employment in agriculture; and a binary measure, which is (1) if the respondent is either the household head or is married to the household head and is (0) otherwise.

### Analytic approach

The first step of the analysis is to establish whether or not there is a relationship between weather pattern change and the prospective ideal fertility of young, childless men and women in Sahelian West Africa, and if the effects of different types of weather vary. In a second set of models, I stratify the sample by climate zone and explore variation by physical capital. In a third set of models, I stratify the sample by gender of the respondent and examine variation by physical capital. In other gender-stratified models, I also predict the ideal number of boys and the ideal number of girls, and investigate the variation in the relationship between these outcomes and weather pattern change by physical capital.

I largely employ Poisson regression models predicting the ideal number of children because the outcome is a count variable, and the mean and the variance of the outcome variable are approximately equal. However, the men’s stratified models predicting the ideal number of boys and the ideal number of girls are negative binomial regression models because the mean is over-dispersed, and is therefore not suitable for Poisson regression. In addition to the controls, all models include birth year fixed effects to account for changing trends across birth cohorts. To account for multiple men and women residing in each DHS cluster, I cluster the standard errors.

## Results

### Descriptive statistics

Table 1 describes the sample of rural, young, childless men and women surveyed by the Demographic and Health Surveys programme in Senegal, Burkina Faso, Mali and Nigeria. In total, the average ideal number of children for this sample is 5.7. When broken down by the sex of the child, the average ideal number of girls is 2.3 and the average ideal number of boys is 2.9. Women make up the majority of the sample, at 71 per cent. The average male respondent is 23.3 years old and the average female respondent is 17.5 years old. A small portion (17 per cent) of the respondents are the household head or are married to the household head. Over half of the sample, 58 per cent, have a primary-level education or more. About one-quarter of the respondents report that their current occupation is in the agricultural sector. Over half of the sample, 56 per cent, live in the equatorial climate zone. In terms of household physical capital, 80 per cent of the respondents live in a household that owns agricultural land, and three-quarters live in a household that owns livestock.

The men and women in this sample were surveyed in 3,724 clusters. In the 10 kilometres surrounding each cluster, the average monthly rainfall was 9.2cm and the average temperature was 27.7 degrees Celsius during the 10-year baseline period. The range of change in temperature was large, between −2.25 and 1.25 standard deviation units, which is equal to −3.4 to 2.5 degrees Celsius. The range of change in precipitation was more limited, between −0.38 and 0.24 standard deviations units, which translates to −3.3 to 2.6 cm of rain.

### Multivariate results

The first goal of the analysis is to establish whether or not there is a relationship between changing weather patterns and the ideal number of children for young, childless men and women in Sahelian West Africa, and to examine the differences by weather type. Model 1 (Table 2) shows the results of the Poisson model predicting the ideal number of children, which indicate that while there is a relationship between weather pattern change and ideal fertility among this sample of young, childless adults, it is confined to the change in temperature. For each one standard deviation unit increase in temperature, the increase in the log of the expected count of the ideal number of children is 0.069 (*p* < .01). This main model also shows that being in the equatorial climate zone has a small, negative effect on the ideal number of children (−0.045 *p* < .001). Both physical capital measures have positive effects on the outcome: household landownership has an effect of 0.047 (*p* < .001) and household livestock ownership has an effect of 0.024 (*p* < .001). Gender is also an important predictor, as being a woman has a negative effect of −0.186 (*p* < .001). These results confirm that climate, physical capital and gender are salient for the formation of the ideal family size.

**Figure 3:**
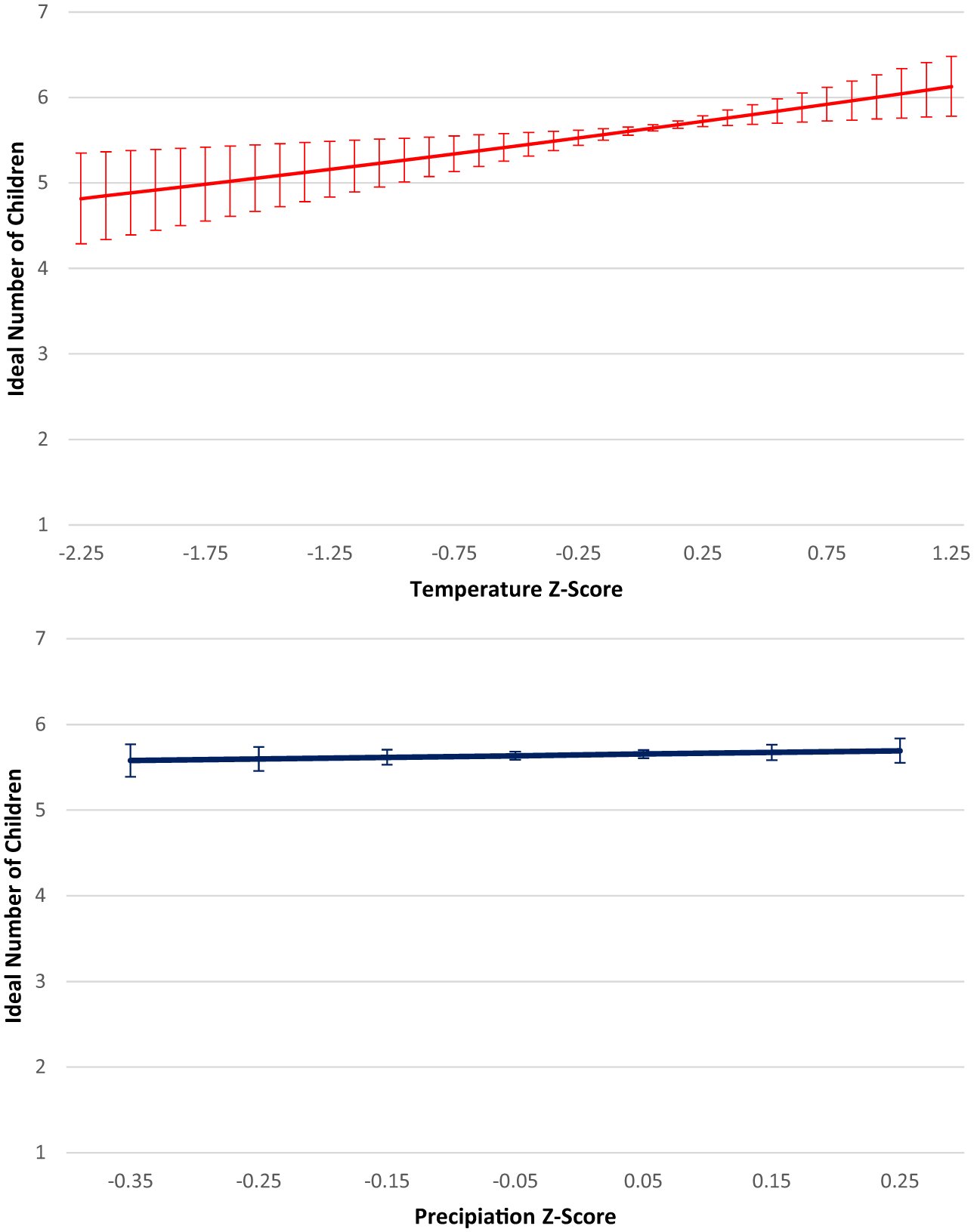
Predicted ideal fertility for childless young adults in Sahelian West Africa across weather change

The top panel of Figure 3 provides the predicted count of the ideal number of children for this model; at about a z-score of zero (average temperature conditions), the predicted ideal number of children is 5.6. Where the change in temperature is negative, reaching a value of −2.25 standard deviation units, the ideal count is 4.8 children. On the opposite end, where the change in temperature is positive, reaching a z-score of 1.25, the ideal count is 6.12 children. In simpler terms, recent hotter temperatures are associated with a higher ideal number of children. The change in precipitation is not a significant predictor of the ideal number of children. In the bottom panel of Figure 3, the relatively flat line shows that the minimal changes in average precipitation do not affect the ideal number of children, which stays steady at about 5.6 children.

#### Climate-stratified models

The next goal of the analysis is to explore variation in the relationship between weather pattern change and the ideal number of children by climate type. The relationship is expected to vary by climate based on the different weather, landscapes and agricultural characteristics of each zone. Tables 3A and 3B present the Poisson models predicting the ideal number of children for each climate zone. Model 2 (Table 3A) shows results for the arid subsample. Temperature z-score has a positive, significant effect on the log of the expected count of the ideal number of children, 0.19 (*p* < .001). Model 3 (Table 3A) shows a small and partially significant relationship between weather pattern change and the ideal number of children for the equatorial subsample. Temperature z-score has a small positive effect, 0.08 (*p* < .05), and precipitation z-score has a positive and marginally significant effect, 0.14 (*p* < .10), on the log of the expected count of the ideal number of children.

**Figure 4:**
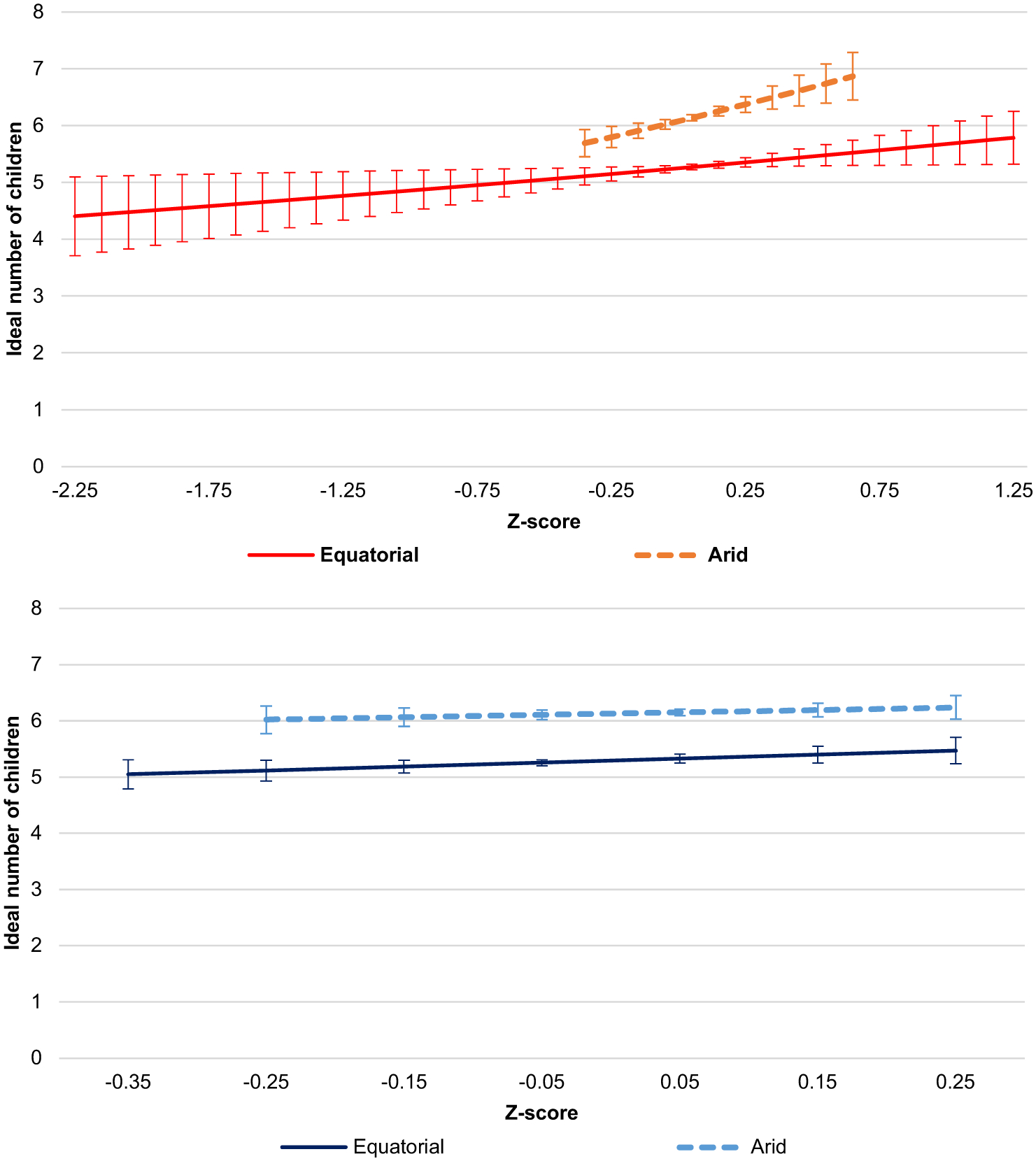
Predicted ideal fertility for childless young adults in Sahelian West Africa by climate zones across weather change

Figure 4 presents the predicted counts of the ideal number of children for each subsample across temperature (top panel) and precipitation (bottom panel) change. These graphs highlight that the ideal is higher in the arid climate. In this climate zone, where a recent increase in average temperatures is reported, there is a significantly higher predicted ideal count, reaching 6.9 children. The bottom panel shows the lack of an effect of precipitation change in the arid climate zone and the very slight positive effect in the equatorial climate zone. These analyses provide support for the expectation that the relationship between weather pattern change and ideal fertility would vary by climate zone; and evidence of the particular effect of temperature change.

Models 4 through 7 (Table 3B) include interactions between the physical capital measures and the weather change measures for each climate sample. While household ownership of livestock affects the ideal number of children (slightly, about 0.02 (*p* < .05) in Models 4 and 5), it does not affect the relationship between weather pattern change and ideal fertility in either climate zone (Models 4 and 5, Table 3B), which is as expected.

**Figure 5:**
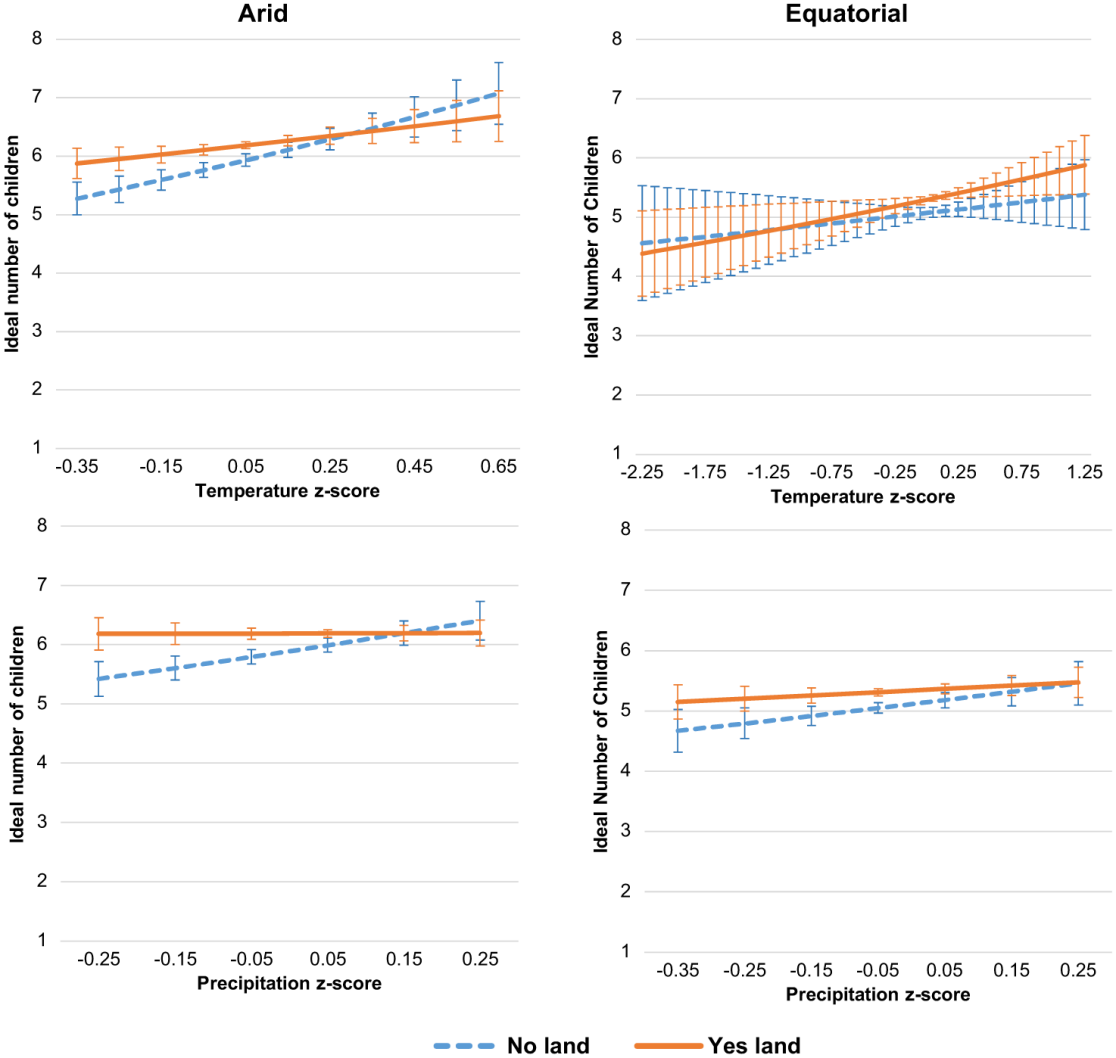
Predicted ideal fertility for childless young adults in Sahelian West Africa by climate zones across weather change by landholding status

Model 6 (Table 3B) includes the interactions with ownership of agricultural land for the arid subsample. The household landownership and temperature z-score interactions and the household landownership and precipitation z-score interactions both have negative effects on the log of the expected count of the ideal number of children of −0.16 (*p* < .01) and −0.33 (*p* < .01), respectively. The main effect of landownership has a positive effect of a small magnitude, 0.06 (*p* < .001), on the log of the expected count of the ideal number of children. Model 7 (Table 3B) also shows small positive main effects of either type of physical capital ownership, but no interaction effect between landownership and either of the z-score measures for the equatorial subsample. The predicted counts from Models 6 and 7 are graphed in Figure 5. In the arid sample (left panels), temperature change has a small positive effect on respondents in households with and without agricultural land, but as temperatures increase to be above average, the predicted counts overlap. Only when temperatures are below average do these two groups show significantly different ideals; at the maximum level, respondents in households with land have a 0.5 higher ideal family size than respondents in landless households. As expected, respondents in landowning households have higher ideal fertility, and their fertility ideals are somewhat more insulated from weather change than those of respondents who do not own land. The bottom panel shows that precipitation change has no effect on the ideal fertility among respondents in households with land. Both types of weather change have a greater effect for men and women in landless households. In the equatorial (right) panels of Figure 5, it is evident that although the two groups display slightly different slopes, there are no significant differences in the ideal number of children by landholding status in the equatorial climate zone.

#### Gender-stratified models

**Figure 6:**
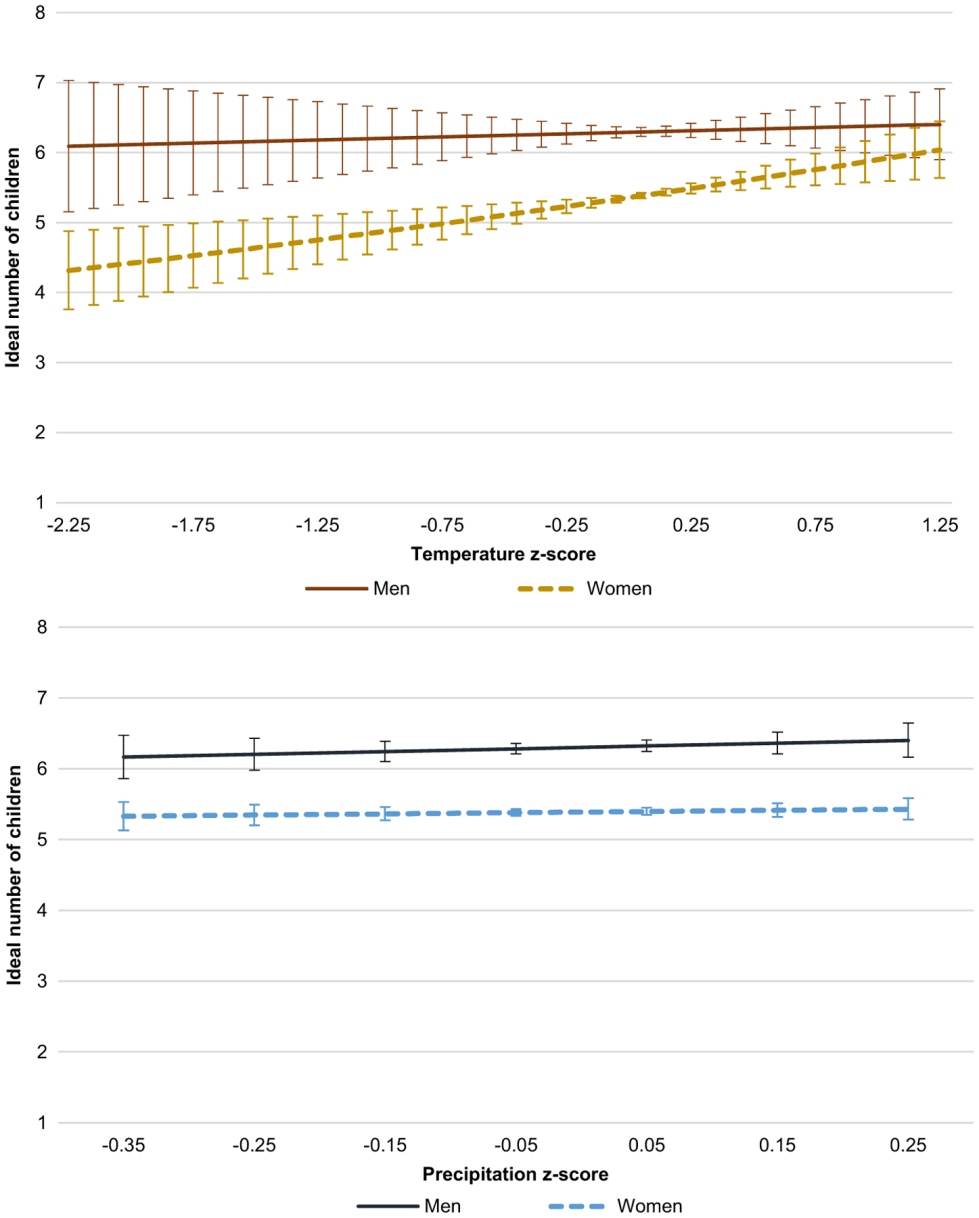
Predicted ideal fertility for childless young adults in Sahelian West Africa by gender across weather change

This set of analyses explores variation in the relationship between weather pattern change and ideal fertility by stratifying the samples by gender (see Table 4). Model 8 (Table 4) predicts the ideal count of children for the men’s subsample. There is no significant effect of weather pattern change on the outcome. There is, however, a significant (*p* < .001) and positive effect of temperature change for women of 0.096, as shown in Model 9. Figure 6 presents the predicted ideal counts for men and women across temperature z-score (top panel) and precipitation z-score (bottom panel). As temperature increases relative to the baseline, the predicted ideal count for women increases, reaching six children at the largest positive deviation in temperature, which is an increase of 0.68 from the predicted count at average conditions. This increase in ideal fertility at the highest temperatures for women makes their ideal similar to that of men, which is not affected by temperature change. In the bottom panel, the graph shows nearly parallel trends for men and women; the ideals of neither group are affected by precipitation change. The difference between men’s and women’s ideals at average conditions is also evident in these graphs, with men’s ideal number of children being approximately one child higher than that of women.

**Figure 7:**
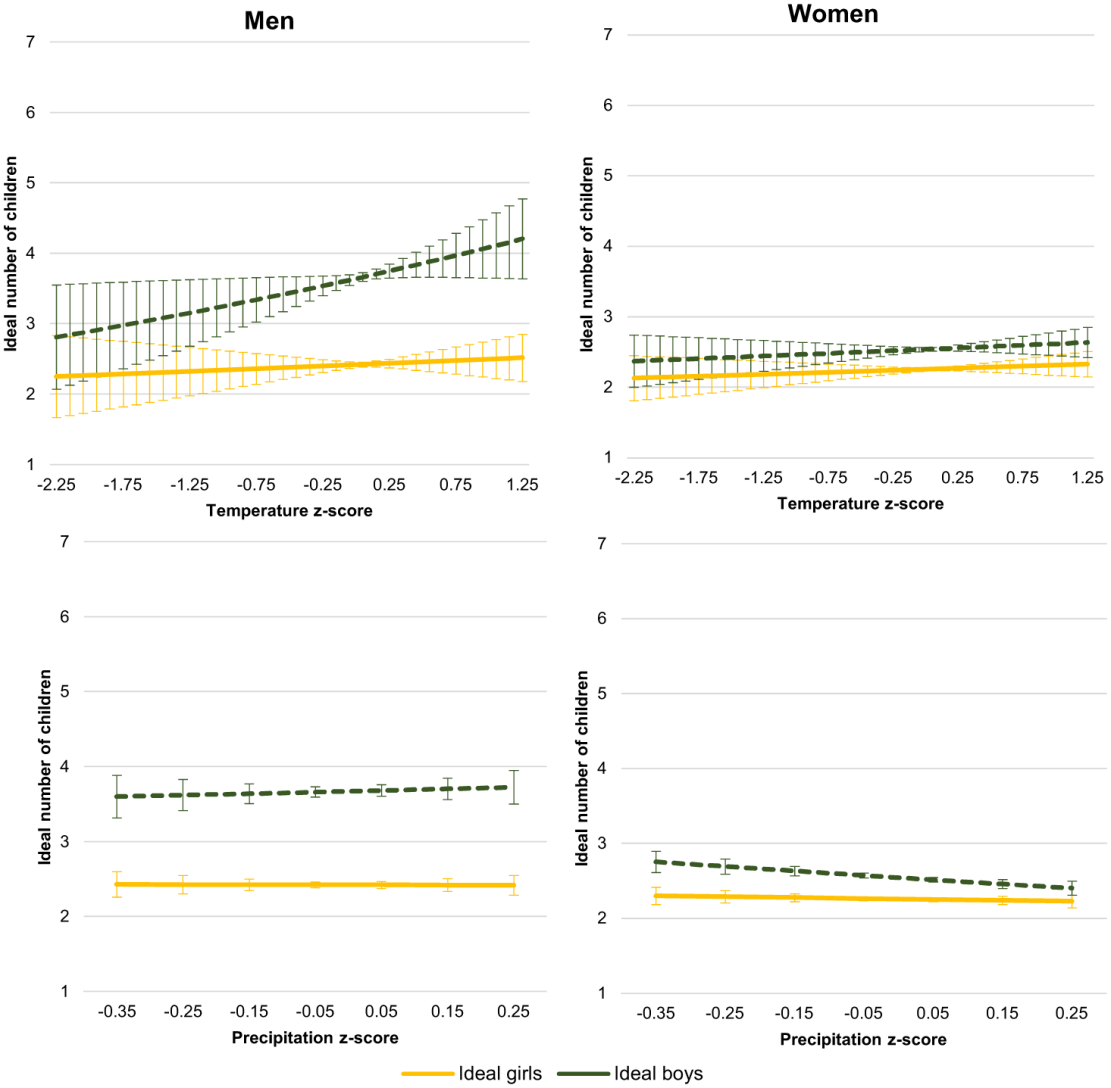
Predicted ideal boys and girls for childless young adults in Sahelian West Africa by gender across weather change

The next set of gender-stratified models predict the ideal number of girls and the ideal number of boys. Table 5 displays the negative binomial regression models predicting each of these outcomes for the men’s sample and Poisson regression models for the women’s sample. For the men’s sample, the ideal number of girls is not affected by changes in weather patterns (Model 10, Table 5), but there is a small positive effect of temperature change on the ideal number of boys, 0.12 (*p* < .05), in Model 11 (Table 5). For the women’s sample, there is no effect of weather pattern change on the ideal number of girls in Model 12 (Table 5), but the ideal number of boys is affected by precipitation change, −0.23 (*p* < .01) in Model 13 (Table 5). When the predicted ideals are graphed, in Figure 7, a preference for boys is evident, especially among men. This pattern holds in most weather conditions, but is especially pronounced for men in hotter conditions. Among men, increasing temperature (top left panel) has a positive effect on the predicted ideal number of boys, reaching 4.2 in the hottest conditions, which is 1.7 children higher than their ideal number of girls under the same temperature conditions. Recent precipitation change has no effect on men’s predicted ideal number of boys or girls (bottom left panel). Among women (right panels), the difference between the ideal number of boys and girls is much narrower, between 0.2 and 0.4. In average temperature conditions (right top panel), women want slightly more boys, but change in either direction adjusts the prediction, making the ideals by gender statistically indistinguishable. As precipitation decreases, the ideal number of boys increases, while the ideal number of girls stays steady (bottom right panel).

**Figure 8:**
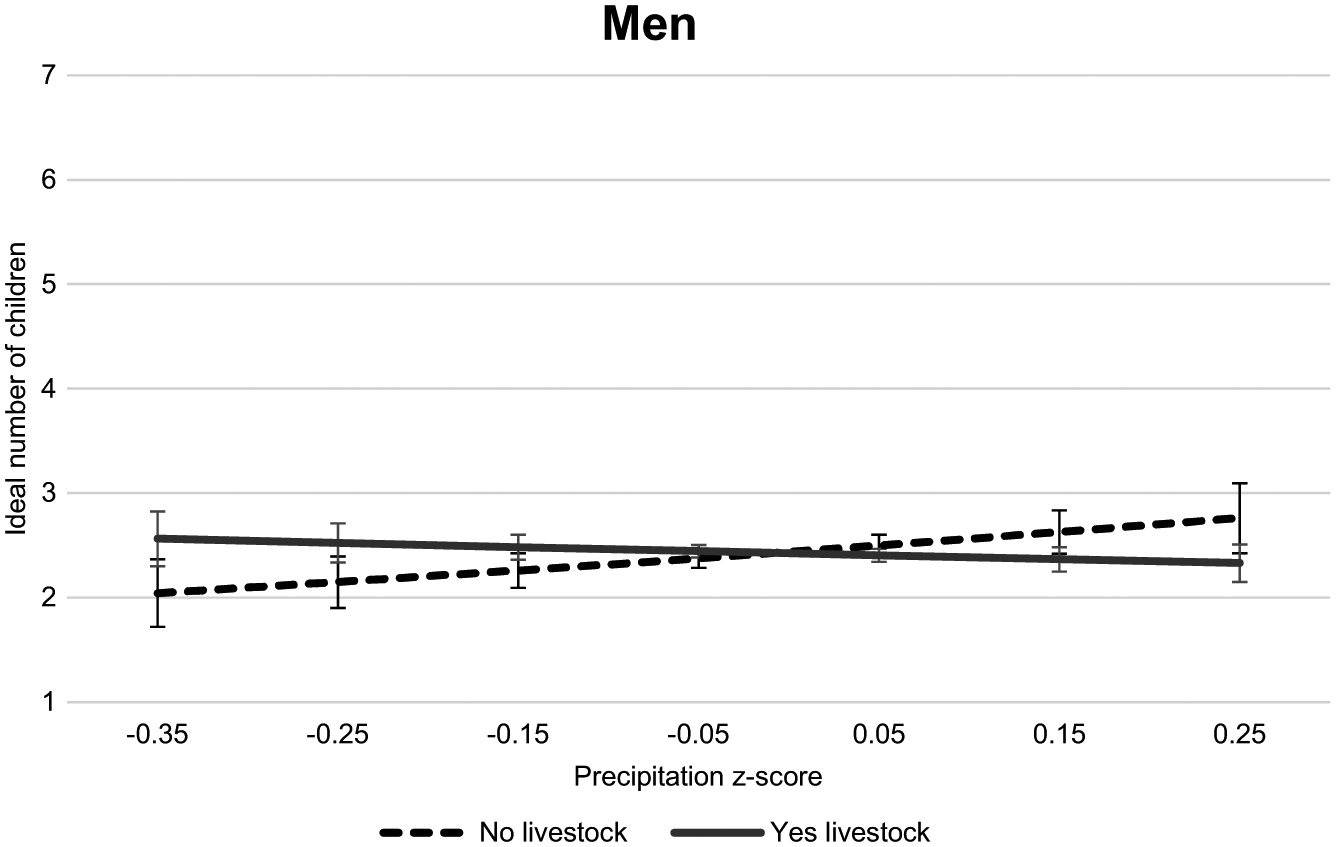
Predicted ideal girls for childless young men in Sahelian West Africa weather change by livestock ownership

The last set of analyses determines whether these differences in the effects of weather pattern change on the ideal number of boys and the ideal number of girls differ by physical capital ownership, which would suggest that the differences could be related to gendered agricultural pursuits or environmental interactions of both adults and children. Table 6A presents the results of negative binomial regression models for the men’s sample, and Table 6B presents the results of Poisson models for the women’s sample, predicting the ideal number of girls and the ideal number of boys, each with land and livestock interactions. Model 14 (Table 6A) shows a negative and significant interaction between the precipitation z-score and livestock ownership in predicting the ideal number of girls for men, −0.66 (*p* < .01). However, when graphed in Figure 8, the opposing effects do not yield the predicted ideals, which are significantly different from one another.^11^ No other model (Model 16 (Table 6A), 18 or 20 (Table 6B)) has a significant interaction between weather pattern change measures and household livestock ownership.

**Figure 9:**
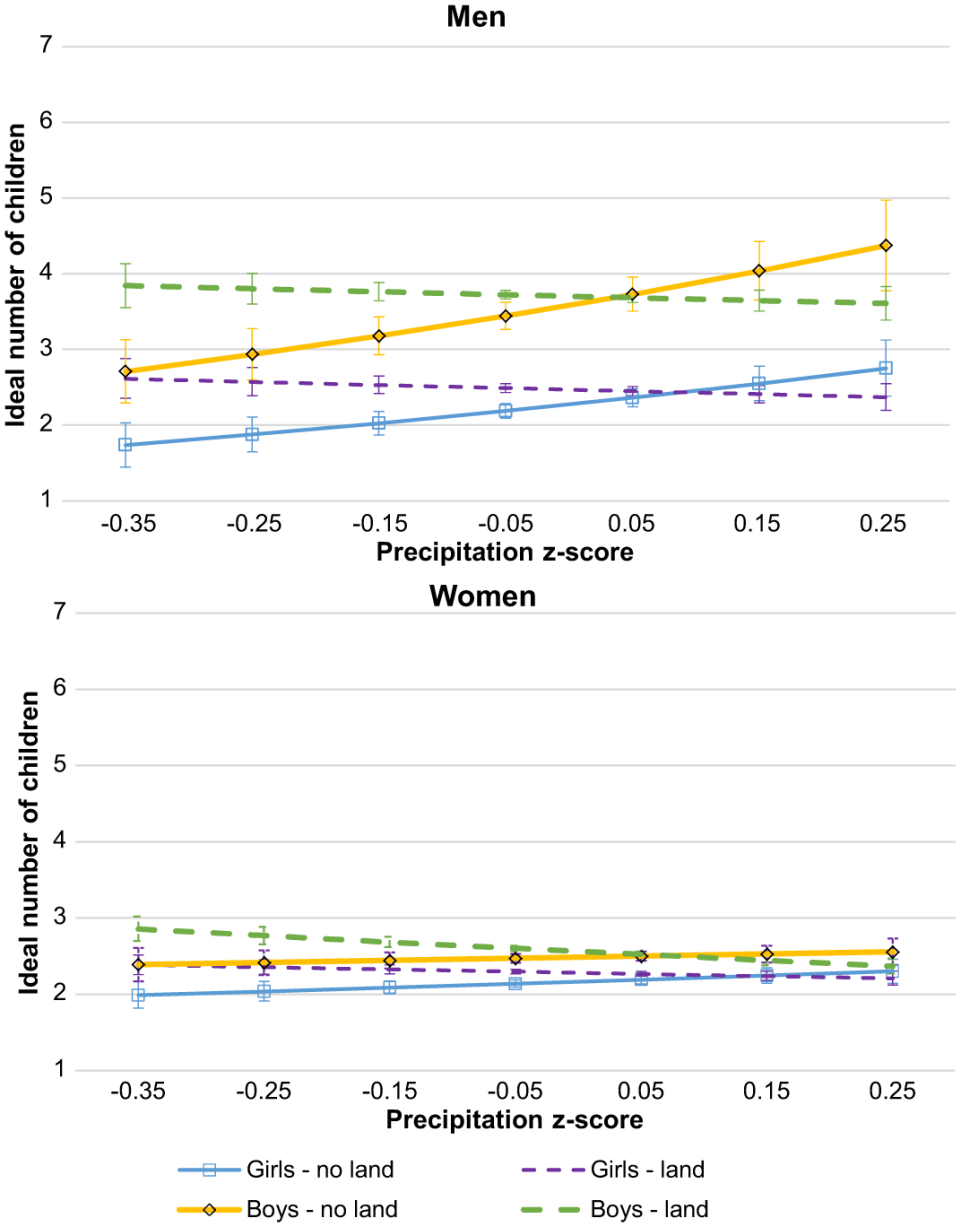
Predicted ideal boys and girls for childless young adults in Sahelian West Africa by gender across weather change by landholding status

In Models 15, 17 (Table 6A), 19 and 21 (Table 6B), interactions between precipitation change and ownership of agricultural land are negative and significant. In the men’s models, Models 15 and 17, the coefficients are approximately −0.9 (*p* < .001); and in the women’s models, Models 19 and 21, the effects are about half that, at −0.37 and −0.43 (*p* < .01). There is no significant interaction effect with the temperature z-score in any model. Figure 9 graphs the predicted counts of boys and girls for respondents in households with and without land in a separate panel for each gender subsample across precipitation change. From these graphs it is apparent that, similar to the land interaction graphs in Figure 5, men and women in landholding households have ideal counts that are negatively affected by increasing precipitation, while their counterparts in landless households have ideal counts that are positively affected by increasing precipitation. Following recent drier conditions, respondents in landholding households have higher ideal counts of boys and girls than those in landless households. Moreover, similar to the graph in the bottom left panel of Figure 7, there is a significant gap between predicted ideals by the gender of the child. As temperature increases, the gap between the ideals by gender widen for respondents without land, with the ideal count of boys rising faster.

In the bottom panel of Figure 9, the graph for women is displayed. There are similar trends between the panels, though the range of ideal counts is much narrower for women than for men. In the driest conditions, the ideal number of girls and boys for women with land is 0.4 children higher than for women without land. As conditions change to be wetter, the predicted counts become almost equal, at about 2.2 girls and about 2.5 boys. Again, a preference for boys is evident, and women with land in dry conditions want the highest number of boys, at 2.9.

## Discussion and conclusion

Populations in West Africa are particularly vulnerable to the effects of climate change because of their dependence on rain-fed subsistence agriculture. The rural livelihoods framework posits that in order to extract a living from their surroundings, rural households grapple with the recent environmental changes by investing in other forms of capital, like human capital. Although fertility is a well-established approach for dealing with environmental change (Brauner-Otto, 2014) or other resource strain (Sobotka et al., 2011), achieving one’s desired fertility is not guaranteed. Therefore, prospective ideal fertility (planned investment in human capital), or the number of children and the composition of the ideal family that childless people want to have, may more clearly represent a response to a change in circumstances.

The first goals of this work were to examine whether and, if so, how the ideal fertility of young, childless adults responds to recent weather changes in Sahelian West Africa, and how this relationship varies by the type of weather. As expected, the impact of temperature change on the formation of ideal family size is particularly large, as the predicted ideal number of total children was shown to increase when conditions are hotter than average among young, childless adults in this region as a whole. A family with six children is considered ideal when temperatures reach their highest positive deviations. In the parlance of the livelihood framework, this suggests that having more children to assist in household production is advantageous in these conditions. The precipitation change during this time period is limited, and thus may not be large enough to garner an effect on ideal fertility for the whole sample.

The next goals of this study were to explore sources of heterogeneity in the relationship between weather pattern change and ideal fertility. In the arid climate, where the temperatures are higher, the ideal number of children is also larger. Increases in average temperatures in the already hottest region push the ideal family size higher, from 5.7 children to 6.9 children. This has worrying implications for the future, because temperatures in this climate zone are expected to continue to rise, reaching up to six degrees Celsius by the end of the century (Lee et al., 2023; Niang et al., 2014). Therefore, ideal fertility may continue to increase along with the warming trend. If individuals in this climate zone achieve this (increasing) ideal fertility level, it may put increased strain on already scarce resources. Weather pattern change has a more muted effect on prospective ideal fertility for young, childless adults in the equatorial climate, including a small positive temperature effect and a marginally significant positive precipitation effect. Although the equatorial climate has more favourable conditions for agriculture, the men and women in this subsample are less agricultural than those the arid subsample, as fewer adults in the equatorial climate zone are formally employed in agriculture or live in households that own land or livestock (bivariate analyses not shown). It is thus not surprising that the arid subsample was found to be more affected by weather pattern change.

Another goal of this work was to examine the variation in these relationships by household physical capital. In the arid climate zone, both temperature and precipitation change were shown to be predictive of ideal fertility by landholding status. Adults in households without agricultural land have fertility ideals that are more affected by weather change than those of adults in households with land. In drier and cooler conditions, adults with land have higher predicted ideals than adults without land; but in wetter and hotter conditions, the two groups have similar ideals. It is likely that *owning* land somewhat tempers the need for additional children following recent weather changes, as posited by the land security hypothesis (Stokes and Schutjer, 1984). This group may also find other ways to deal with environmental stressors, such as some form of temporary or seasonal labour migration (Call and Gray, 2020; Weinreb et al., 2020). In this analysis, no particular effect of livestock ownership on the relationship between weather pattern change and fertility ideals was detected, even though livestock ownership is common in the arid climate zone.

The gender of the adult respondent was found to be a salient source of heterogeneity in the weather change and ideal fertility relationship. As expected, in baseline conditions, men have a (one child) higher ideal number of children than women. In terms of how men and women respond to weather change, only women’s ideal number of children is affected by temperature change, such that in hotter conditions their ideal is higher, nearing men’s higher ideal. This finding suggests that weather change may have a particular effect on the natural capital that women interact with, i.e., the resources that they use (likely for household subsistence); and that the increased anticipated labour can be, to some degree, outsourced to children. Increased time to collect resources has been shown to have a positive effect on desired family size for women in Nepal (Brauner-Otto and Axinn, 2017). Having additional children in times of environmental stress can contribute to a cycle of further environmental and household strain, termed the vicious circle model (Biddlecom et al., 2005; Filmer and Pritchett, 2002; Lutz and Scherbov, 2000). Such findings provide further evidence that women’s solutions to environmental stress tend to be focused on the short term, and are often not the most sustainable practices, because women are disadvantaged in their access to information, credit, natural capital and other resources (Jin et al., 2015; Lambrou and Piana, 2006; Masika, 2002; Silvestri et al., 2012; Terry, 2009).

This work also explored the gender composition of the ideal number of children. A son preference is evident, especially among men. The gender composition ideals of men are impacted by temperature change. When conditions are cooler than average, men’s ideals by child gender are statistically similar; whereas when temperatures rise, men’s ideal number of boys increases significantly, while their ideal number of girls increases only slightly. There is no significant effect of temperature change on women’s ideal gender composition of children; while women want a higher average number of boys, this number is steady across temperature deviations. There is a small increase in women’s ideal number of boys when conditions are drier than average. Taken together with the findings related to the ideal total number of children by the respondent’s gender, this shows that while women want more children in hotter conditions, this preference does not translate to a distinctly unbalanced gender composition of children. Men, on the other hand, do not prefer a higher total number of children in hotter conditions, but do prefer significantly more boys. Men get more specific about the gender composition of their ideal number of children in hotter conditions. If the number of boys increases and the number of girls stays steady, then the ideal number for which gender does not matter goes down. Thus, gender appears to be more salient in these conditions for the person who is likely to be in charge of fertility decision-making in the near term (Ezeh, 1993; Bankole, 1995). Men’s desire to have more boys may imply a need for labour after labour-related differentiation by gender has occurred, usually in the early teens. Moreover, men could see boys as providing a longer-term payoff in terms of their future wage-earning potential and their ability support to their parents. Thus, the gender composition of men’s fertility ideals appears to reflect more long-term concerns, rather than the general, short-term need for children that women’s fertility ideals express.

This long-term orientation is confirmed in Figure 9. For this sample of rural men and women with strong connections to agriculture, if an adverse weather-generated preference for boys suggests that boys’ labour is more highly valued, and especially in these conditions, then it would be logical to expect to observe a particular effect of weather change on the ideal number of boys by landholding, since crop cultivation is labour-intensive. The findings indicate that there is indeed a stronger preference for boys in landholding households, but the trend in the ideal count parallels that for girls and is similar to the effects by landholding when predicting the ideal total number of children. Therefore, it is possible that men anticipate these weather pattern changes continuing in the future, and expect higher returns and more old-age security from their adult male children. By contrast, short-term environmental concerns, which may be more pronounced for women, necessitate having more children to meet the household’s labour needs *before* work-related differentiation by gender in children occurs.

These results come with some limitations. First, to minimise rationalisation bias in the ideal fertility outcomes, I removed young adults with children from the analytical sample, who account for about 40 per cent of young adults in rural areas. I surmised that if I could measure the fertility goals of this group before they had children, their inclusion would sharpen the effects by temperature, because the majority of young adults with children are women and live in the arid climate zone, and they are also more likely to own livestock and land. Given this limitation, I am only able to generalise to young adults on the precipice of their reproductive careers. The use of longitudinal data could overcome this limitation. Second, the DHS are nationally representative household surveys that are well-regarded in research. More commonly, the women’s surveys are used. I made use of both the women’s and the men’s surveys. The men’s sample may be more selective than the women’s sample. The DHS interview men in only one-third of households that are selected for female questionnaires. In addition, many men could be at work or away from the area (migration for work is common in the region), while women are more likely to be engaged in labour near their home. Despite these differences, using many surveys across the region gave me a sizeable subsample with which to work. Third, this study focuses on four specific countries. I selected these countries (from among those with appropriate DHS data) because they encompass both arid and equatorial climates. However, restricting my focus to them prevented me from addressing more generally the effects of climate change on other parts of West Africa. Fourth, I included the measures of physical capital and assumed that each is used to support the household’s livelihood. However, I was unable to verify this assumption because the DHS do not collect information on how households currently use their land. Lastly, and relatedly, livestock ownership was not found to be particularly relevant for the relationship of interest. While I posit that this finding reflects the reduced labour needs in animal husbandry and adaptation to weather change, it is possible that it points to a sampling bias due to the remote location and seasonality of pastoralism, as those who engage in this practice may be away for significant periods of time, often in sparsely populated areas of the region. The mobility of pastoralists hinders data collection by traditional means (Randall, 2015), but sampling over many months, as the DHS programme does, may reduce this concern.

Climate change is an issue affecting many societies across the globe. The countries of West Africa may be especially vulnerable due to their reliance on subsistence and rain-fed agriculture and their fast-growing populations. This study has provided an examination of how recent changes in precipitation and temperature affect the prospective ideal fertility of rural, young, childless adults in the Sahelian region of West Africa, and variations therein. The findings reveal that changes in weather patterns inform young people’s ideal fertility calculus in context-dependent ways. Gender is particularly salient in this relationship. The gendered ways in which individuals interact with their changing environment affect the ways they plan their families. Climate and physical capital also matter for this relationship. The different weather pattern changes and the distinct baseline environmental conditions people operate in affect the meaning of weather pattern change in different climate zones. Household physical capital and livelihood strategies also inform the ways weather change is translated into fertility ideals. These findings nuance our understanding of the climate-fertility relationship in the region, and shed more light on how people are making intimate family decisions in response to the climate crisis. Given the close correlation between ideal and actual fertility in West Africa and the expected changes in temperature and rainfall patterns, weather-related changes in ideal fertility may signal changes in actual fertility. Understanding the factors relevant to fertility decision-making can make it easier to support individuals and households in achieving their preferences or adapting to new conditions in sustainable ways.

## Figures and Tables

**Table 1:** Descriptive statistics of young, childless adults and the weather patterns surrounding the Demographic and Health Survey clusters in which they reside

Individual-level measures (*n* = 33,732)	Mean/Per cent	SD	Min	Max
Ideal number of children	5.7	2.4	0	10
Ideal number of girls	2.3	1.6	0	10
Ideal number of boys	2.9	2.0	0	10
Men	29.3%	0.5		
Women	70.7%	0.5		
Age – men	23.3	3.4	19	31
Age – women	17.5	2.2	15	23
Completed (at least) primary school	57.5%	0.5		
Household head or married to head	17.3%	0.4		
Formally employed in agriculture	26.0%	0.4		
Climate zone				
Arid climate	44.4%	0.5		
Equatorial climate	55.6%	0.5		
Physical capital				
Household owns agricultural land	79.8%	0.4		
Household owns livestock	73.8%	0.4		
Cluster-level measures (*n* = 3,724)	Mean	SD	Min	Max
Weather measures				
Mean baseline precipitation (cm)	9.2	4.5	1.7	22.0
Mean baseline temperature (C°)	27.7	1.1	20.8	30.7
Weather z-scores				
Z-score – temperature	0.1	0.2	−2.3	1.2
Z-score – precipitation	0.0	0.1	−0.4	0.2

**Table 2:** Poisson model results predicting the ideal number of children for young, childless adults with clustered standard errors

	Model 1		
	Coef	SE	Sig
Age	0.015	0.007	*
Woman (ref: Man)	−0.186	0.008	***
Completed (at least) primary school	−0.154	0.007	***
Household head or married to head	0.080	0.007	***
Formally employed in agriculture	0.023	0.006	***
Equatorial climate zone (ref: arid climate)	−0.045	0.012	***
Physical capital			
Household owns agricultural land (ref: no land)	0.047	0.007	***
Household owns livestock (ref: no livestock)	0.024	0.006	***
Weather measures			
Mean baseline precipitation (cm)	−0.006	0.002	***
Standard deviation baseline precipitation (cm)	0.016	0.002	***
Mean baseline temperature (C°)	−0.023	0.004	***
Standard deviation baseline temperature (C°)	0.108	0.008	***
Weather z-scores			
Z-score – temperature	0.069	0.024	**
Z-score – precipitation	0.034	0.049	
Constant	1.849	0.195	***
*N*	33732		

Notes: Model includes three squared terms to account for non-linearities: age^2^, z-score temperature^2^, and z-score precipitation^2^ and birth year fixed effects; clustered standard errors in second column.

+ *p* < 0.10,

* *p* < 0.05,

** *p* < 0.01,

*** *p* < 0.001

**Table 3A:** Poisson model results predicting the ideal number of children for young, childless adults with clustered standard errors in climate-stratified samples

	Arid			Equatorial		
	Model 2			Model 3		
	Coef	SE	Sig	Coef	SE	Sig
Age	0.033	0.010	***	0.007	0.009	
Woman (ref: Man)	−0.253	0.011	***	−0.134	0.010	***
Completed (at least) primary school	−0.141	0.009	***	−0.150	0.011	***
Household head or married to head	0.117	0.010	***	0.061	0.009	***
Formally employed in agriculture	0.016	0.009	+	0.021	0.009	*
Physical capital						
Household owns agricultural land (ref: no land)	0.045	0.010	***	0.046	0.009	***
Household owns livestock (ref: no livestock)	0.022	0.010	*	0.022	0.008	**
Weather measures						
Mean baseline precipitation (cm)	−0.017	0.011		0.006	0.002	*
Standard deviation baseline precipitation (cm)	0.029	0.010	**	0.010	0.003	***
Mean baseline temperature (C°)	−0.018	0.005	***	−0.018	0.007	**
Standard deviation baseline temperature (C°)	0.076	0.011	***	0.214	0.020	***
Weather z-scores						
Z-score – temperature	0.188	0.052	***	0.078	0.035	*
Z-score – precipitation	0.071	0.074		0.135	0.078	+
Constant	1.969	0.215	***	1.498	0.249	***
*N*	14962			18770		

Notes: Models include three squared terms to account for non-linearities: age^2^, z-score temperature^2^, and z-score precipitation^2^ and birth year fixed effects; clustered standard errors in second column.

+ *p* < 0.10,

* *p* < 0.05,

** *p* < 0.01,

*** *p* < 0.001

**Table 3B:** Poisson model results predicting the ideal number of children for young, childless adults with clustered standard errors and physical capital interactions in climate-stratified samples

	Arid			Equatorial			Arid			Equatorial		
	Model 4			Model 5			Model 6			Model 7		
	Coef	SE	Sig	Coef	SE	Sig	Coef	SE	Sig	Coef	SE	Sig
Age	0.033	0.010	***	0.007	0.009		0.033	0.010	**	0.007	0.009	
Woman (ref: Man)	−0.253	0.012	***	−0.134	0.010	***	−0.252	0.011	***	−0.134	0.010	***
Completed (at least) primary school	−0.141	0.009	***	−0.151	0.011	***	−0.140	0.009	***	−0.150	0.011	***
Household head or married to head	0.117	0.010	***	0.061	0.009	***	0.117	0.010	***	0.061	0.009	***
Formally employed in agriculture	0.016	0.009	+	0.021	0.009	*	0.016	0.009	+	0.021	0.009	*
Physical capital												
Household owns agricultural land (ref: no land)	0.045	0.010	***	0.046	0.009	***	0.057	0.011	***	0.040	0.010	***
Household owns livestock (ref: no livestock)	0.026	0.011	*	0.019	0.009	*	0.023	0.010	*	0.022	0.008	**
Weather measures												
Mean baseline precipitation (cm)	−0.017	0.011		0.006	0.002	*	−0.017	0.011		0.006	0.002	*
Standard deviation baseline precipitation (cm)	0.029	0.010	**	0.010	0.003	***	0.029	0.010	**	0.010	0.003	***
Mean baseline temperature (C°)	−0.018	0.005	***	−0.018	0.007	**	−0.019	0.005	***	−0.018	0.007	**
Standard deviation baseline temperature (C°)	0.076	0.011	***	0.214	0.020	***	0.079	0.011	***	0.213	0.020	***
Weather z-scores												
Z-score – temperature	0.232	0.073	**	0.056	0.039		0.293	0.0626	***	0.047	0.047	
Z-score – precipitation	0.123	0.118		0.164	0.102		0.333	0.102	**	0.26	0.117	*
Interactions												
Z-score – temperature * Household owns livestock (ref: no livestock)	−0.053	0.067		0.031	0.037							
Z-score – precipitation * Household owns livestock (ref: no livestock)	−0.062	0.114		−0.042	0.113							
Z-score – temperature * Household owns ag. land (ref: no land)							−0.164	0.061	**	0.037	0.042	
Z-score – precipitation * Household owns ag. land (ref: no land)							−0.328	0.106	**	−0.158	0.122	
Constant	1.966	0.215	***	1.498	0.249	***	1.963	0.214	***	1.500	0.250	***
*N*	14962			18770			14962			18770		

Notes: Models include three squared terms to account for non-linearities: age^2^, z-score temperature^2^, and z-score precipitation^2^ and birth year fixed effects; clustered standard errors in second column.

+ *p* < 0.10,

* *p* < 0.05,

** *p* < 0.01,

*** *p* < 0.001

**Table 4:** Poisson model results predicting the ideal number of children for young, childless adults with clustered standard errors in gender-stratified samples

	Men’s			Women’s		
	Model 8			Model 9		
	Coef	SE	Sig	Coef	SE	Sig
Age	0.005	0.018		0.089	0.020	***
Completed (at least) primary school	−0.162	0.011	***	−0.144	0.008	***
Household head or married to head	0.035	0.011	**	0.125	0.009	***
Formally employed in agriculture	0.033	0.009	***	0.000	0.008	
Equatorial climate (ref: arid)	−0.058	0.018	***	−0.043	0.013	**
Physical capital						
Household owns agricultural land (ref: no land)	0.047	0.012	***	0.046	0.008	***
Household owns livestock (ref: no livestock)	0.010	0.010		0.027	0.007	***
Weather measures						
Mean baseline precipitation (cm)	−0.017	0.002	***	−0.002	0.002	
Standard deviation baseline precipitation (cm)	0.017	0.003	***	0.015	0.002	***
Mean baseline temperature (C°)	−0.013	0.006	*	−0.024	0.004	***
Standard deviation baseline temperature (C°)	0.099	0.011	***	0.095	0.009	***
Weather z-scores						
Z-score – temperature	0.014	0.034		0.096	0.029	***
Z-score – precipitation	0.063	0.072		0.032	0.055	
Constant	2.007	0.299	***	1.362	0.223	***
*N*	9899			23833		

Notes: Models include three squared terms to account for non-linearities: age^2^, z-score temperature^2^, and z-score precipitation^2^ and birth year fixed effects; clustered standard errors in second column.

+ *p* < 0.10,

* *p* < 0.05,

** *p* < 0.01,

*** *p* < 0.001

**Table 5:** Negative binomial regression model results predicting the ideal number of girls and boys for the men’s sample and Poisson model results predicting the ideal number of girls and boys for the women’s sample with clustered standard errors

	Men						Women					
	Ideal number of girls			Ideal number of boys			Ideal number of girls			Ideal number of boys		
	Model 10			Model 11			Model 12			Model 13		
	Coef	SE	Sig	Coef	SE	Sig	Coef	SE	Sig	Coef	SE	Sig
Age	0.018	0.036		0.030	0.032		0.109	0.032	***	0.158	0.029	***
Completed (at least) primary school	−0.160	0.018	***	−0.203	0.018	***	−0.106	0.011	***	−0.116	0.011	***
Household head or married to head	0.023	0.019		0.013	0.020		0.087	0.015	***	0.063	0.015	***
Formally employed in agriculture	0.055	0.016	***	0.030	0.016	+	0.018	0.012		0.037	0.012	**
Equatorial climate (ref: arid)	−0.067	0.023	**	−0.071	0.028	*	−0.075	0.019	***	−0.146	0.019	***
Physical capital												
Household owns agricultural land (ref: no land)	0.093	0.021	***	0.042	0.020	*	0.054	0.012	***	0.032	0.011	**
Household owns livestock (ref: no livestock)	0.003	0.020		0.006	0.018		0.028	0.011	*	0.040	0.011	***
Weather measures												
Mean baseline precipitation (cm)	−0.018	0.004	***	−0.028	0.004	***	−0.001	0.002		−0.019	0.002	***
Standard deviation baseline precipitation (cm)	0.031	0.005	***	0.028	0.005	***	0.013	0.003	***	0.023	0.003	***
Mean baseline temperature (C°)	0.000	0.009		−0.016	0.010		−0.001	0.007		0.019	0.007	**
Standard deviation baseline temperature (C°)	0.094	0.017	***	0.102	0.020	***	0.018	0.014		−0.091	0.013	***
Weather z-scores												
Z-score – temperature	0.032	0.057		0.115	0.058	*	0.026	0.033		0.030	0.035	
Z-score – precipitation	−0.006	0.103		0.057	0.115		−0.055	0.073		−0.228	0.075	**
Constant	0.498	0.639		1.534	0.549	**	0.039	0.349		−0.449	0.336	
/Inalpha	−2.169	0.062	***	−1.988	0.066	***						
alpha	0.114			0.137								
*N*	9879			9879			23607			23607		

Notes: Models include three squared terms to account for non-linearities: age^2^, z-score temperature^2^, and z-score precipitation^2^ and birth year fixed effects; clustered standard errors in second column.

+ *p* < 0.10,

* *p* < 0.05,

** *p* < 0.01,

*** *p* < 0.001

**Table 6A:** Negative binomial regression model results predicting the ideal number of girls and boys for the men’s sample with physical capital interaction and clustered standard errors

	Men											
	Ideal number of girls						Ideal number of boys					
	Model 14			Model 15			Model 16			Model 17		
	Coef	SE	Sig	Coef	SE	Sig	Coef	SE	Sig	Coef	SE	Sig
Age	0.018	0.038		0.020	0.038		0.031	0.032		0.033	0.032	
Completed (at least) primary school	−0.160	0.021	***	−0.159	0.021	***	−0.203	0.018	***	−0.202	0.018	***
Household head or married to head	0.023	0.023		0.024	0.023		0.014	0.020		0.014	0.020	
Formally employed in agriculture	0.054	0.018	**	0.054	0.018	**	0.029	0.016	+	0.029	0.016	+
Equatorial climate (ref: arid)	−0.070	0.032	*	−0.069	0.032	*	−0.073	0.028	*	−0.073	0.028	**
Physical capital												
Household owns agricultural land (ref: no land)	0.094	0.023	***	0.077	0.024	**	0.042	0.020	*	0.029	0.021	
Household owns livestock (ref: no livestock)	0.002	0.022		0.002	0.020		−0.004	0.020		0.005	0.018	
Weather measures												
Mean baseline precipitation (cm)	−0.018	0.004	***	−0.018	0.004	***	−0.027	0.004	***	−0.027	0.004	***
Standard deviation baseline precipitation (cm)	0.031	0.006	***	0.031	0.006	***	0.027	0.005	***	0.028	0.005	***
Mean baseline temperature (C°)	0.000	0.011		0.000	0.011		−0.015	0.010		−0.015	0.010	
Standard deviation baseline temperature (C°)	0.094	0.022	***	0.097	0.022	***	0.101	0.020	***	0.106	0.020	***
Weather z-scores												
Z-score – temperature	0.107	0.115		−0.041	0.115		0.026	0.098		0.061	0.109	
Z-score – precipitation	0.502	0.230	*	0.766	0.249	**	0.199	0.201		0.797	0.214	***
Interactions												
Z-score – temperature * Household owns livestock (ref: no livestock)	−0.105	0.137					0.122	0.120				
Z-score – precipitation * Household owns livestock (ref: no livestock)	−0.662	0.257	**				−0.180	0.222				
Z-score – temperature * Household owns ag. land (ref: no land)				0.092	0.128					0.067	0.126	
Z-score – precipitation * Household owns ag. land (ref: no land)				−0.931	0.272	***				−0.903	0.230	***
Constant	0.508	0.586		0.481	0.586		1.541	0.550	**	1.512	0.555	**
/lnalpha	−2.173	0.098	***	−2.176	0.098	***	−1.989	0.066	***	−1.995	0.066	***
alpha	0.114			0.113			0.137			0.136		
*N*	9879			9879			9879			9879		

Notes: Models include three squared terms to account for non-linearities: age^2^, z-score temperature^2^, and z-score precipitation^2^ and birth year fixed effects; clustered standard errors in second column.

+ *p* < 0.10,

* *p* < 0.05,

** *p* < 0.01,

*** *p* < 0.001

**Table 6B:** Poisson regression model results predicting the ideal number of girls and boys for the women’s sample with physical capital interaction and clustered standard errors

	Women’s											
	Ideal girls						Ideal boys					
	Model 18			Model 19			Model 20			Model 21		
	Coef	SE	Sig	Coef	SE	Sig	Coef	SE	Sig	Coef	SE	Sig
Age	0.108	0.032	***	0.108	0.032	***	0.157	0.029	***	0.156	0.029	***
Completed (at least) primary school	−0.105	0.011	***	−0.105	0.011	***	−0.116	0.011	***	−0.115	0.011	***
Household head or married to head	0.087	0.015	***	0.087	0.015	***	0.063	0.015	***	0.063	0.015	***
Formally employed in agriculture	0.019	0.012		0.019	0.012		0.038	0.012	**	0.039	0.012	**
Equatorial climate (ref: arid)	−0.075	0.019	***	−0.075	0.019	***	−0.146	0.019	***	−0.146	0.019	***
Physical capital												
Household owns agricultural land (ref: no land)	0.054	0.012	***	0.059	0.014	***	0.032	0.011	**	0.037	0.013	**
Household owns livestock (ref: no livestock)	0.035	0.013	**	0.029	0.011	*	0.046	0.012	***	0.041	0.011	***
Weather measures												
Mean baseline precipitation (cm)	−0.001	0.002		−0.001	0.002		−0.019	0.002	***	−0.019	0.002	***
Standard deviation baseline precipitation (cm)	0.013	0.003	***	0.013	0.003	***	0.023	0.003	***	0.023	0.003	***
Mean baseline temperature (C°)	−0.001	0.007		−0.001	0.007		0.018	0.007	**	0.019	0.007	**
Standard deviation baseline temperature (C°)	0.018	0.014		0.019	0.014		−0.091	0.013	***	−0.090	0.013	***
Weather z-scores												
Z-score – temperature	0.113	0.075		0.111	0.088		0.107	0.070		0.120	0.092	
Z-score – precipitation	0.057	0.129		0.243	0.128	+	−0.173	0.126		0.113	0.131	
Interactions												
Z-score – temperature * Household owns livestock (ref: no livestock)	−0.116	0.087					−0.102	0.080				
Z-score – precipitation * Household owns livestock (ref: no livestock)	−0.148	0.139					−0.074	0.137				
Z-score – temperature * Household owns ag. land (ref: no land)				−0.110	0.101					−0.117	0.105	
Z-score – precipitation * Household owns ag. land (ref: no land)				−0.370	0.136	**				−0.425	0.139	**
Constant	0.044	0.349		0.035	0.348		−0.446	0.336		−0.455	0.335	
*N*	23607			23607			23607			23607		

Notes: Models include three squared terms to account for non-linearities: age^2^, z-score temperature^2^, and z-score precipitation^2^ and birth year fixed effects; clustered standard errors in second column.

+ *p* < 0.10,

* *p* < 0.05,

** *p* < 0.01,

*** *p* < 0.00

## Footnotes

1 A livelihood zone is a geographic delineation of an area where a specific livelihood is dominant based on land use.

2 The sample includes surveys from Burkina Faso (2010), Mali (2012, 2018), Nigeria (2008, 2013, 2018) and Senegal (2010–2011, 2012), and continuous DHS each year from 2014 through 2017 (Boyle et al., 2022).

3 To protect respondents’ anonymity there is a random displacement of the location of the cluster location. Rural cluster displacement is up to 5km (and in rare cases (less than one per cent of cases) rural cluster displacement is up to 10km) (Burgert et al., 2013).

4 This limitation reflects the potential for pregnancies to be affected by weather change, particularly extreme heat (Barreca et al., 2018). This limitation is only for women; information on pregnancy terminations or losses are not captured for men in the DHS samples used here.

5 I do not restrict the sample to those who have resided in the cluster location since the beginning of the baseline period because even relative newcomers to the area can be affected by the household resource effects of weather pattern change. It is mainly land and natural capital that are changed, while any effects on individuals are indirect. Even those who are not directly tied to any agricultural production still experience household resource strain because the local economy is driven by local agricultural production.

6 I only include numeric responses because in the majority of surveys used here the non-numeric responses to this item are reported as one non-numeric response, rather than parsed out to categories such as “up to God/Allah” or “fatalistic/any number”, which would make recoding them as numeric values, as is sometimes common in the literature, difficult, and perhaps misleading in this case.

7 The question ends with “and for how many would the sex not matter?” This part of the question is not included in this study.

8 The baseline mean measures for temperature and precipitation are the mean of the annual means for each of the 10 years. The baseline standard deviation measures for temperature and precipitation are the mean of standard deviations for each of the 10 years.

9 The DHS do not always distinguish between self-employment in agriculture and being an agricultural employee, or between types of agricultural livelihoods (fisherman, farmer, pastoralist). Subsistence agriculture is a common household practice even if some household members are employed in another sector. This is not captured in the DHS; therefore, I use ownership of livestock and agricultural land to serve as a proxy for these livelihoods. While it is not known how actively these assets support the household, ownership of such assets in rural areas may influence the extent to which weather change affects individuals’ future plans.

10 I focus on primary-level education because this level would be completed before the “recent” period of interest for even the youngest respondents in the sample, which avoids the possibility that recent weather pattern change affected the respondents’ recent educational attainment.

11 This difference – between the significant interaction in Model 14 and the overlapping confidence intervals in Figure 8 – could reflect that the interaction term is capturing differences only at one point in the precipitation change distribution, and that confidence intervals of predicted values do not directly translate to p-values stated in regression outputs.
